# The γ-Core Motif Peptides of Plant AMPs as Novel Antimicrobials for Medicine and Agriculture

**DOI:** 10.3390/ijms24010483

**Published:** 2022-12-28

**Authors:** Marina P. Slezina, Ekaterina A. Istomina, Tatyana V. Korostyleva, Tatyana I. Odintsova

**Affiliations:** Vavilov Institute of General Genetics RAS, 119333 Moscow, Russia

**Keywords:** antimicrobial peptides (AMPs), γ-core motif, synthetic γ-core peptides, antimicrobial activity, structure-function relationship, novel anti-infective agents

## Abstract

The γ-core motif is a structural element shared by most host antimicrobial peptides (AMPs), which is supposed to contribute to their antimicrobial properties. In this review, we summarized the available data on the γ-core peptides of plant AMPs. We describe γ-core peptides that have been shown to exhibit inhibitory activity against plant and human bacterial and fungal pathogens that make them attractive scaffolds for the development of novel anti-infective agents. Their advantages include origin from natural AMP sequences, broad-spectrum and potent inhibitory activity, and cost-effective production. In addition, some γ-core peptides combine antimicrobial and immunomodulatory functions, thus broadening the spectrum of practical applications. Some act synergistically with antimycotics and fungicides, so combinations of peptides with conventionally used antifungal agents can be suggested as an effective strategy to reduce the doses of potentially harmful chemicals. The presented information will pave the way for the design of novel antimicrobials on the basis of γ-core motif peptides, which can find application in medicine and the protection of crops from diseases.

## 1. Introduction

Antimicrobial peptides (AMPs) play a crucial role in diverse biological processes. They are universal participants of the defense system of all living beings forming the basis of innate immunity [1]. They also preserve the composition and abundance of the beneficial microbiome [2]. The occurrence of AMPs in evolutionarily distant groups of organisms demonstrates their importance as effectors of immunity mechanisms and modulators of host-microbe interactions. AMPs are structurally and functionally diverse both within and across species. Each genome harbors genes encoding from five to ten peptide families [3]. Every species contains a unique, specific pool of AMPs tuned to protect the organism against microbes and to establish interactions with beneficial microorganisms [1]. Plants usually possess more AMPs than other organisms [4]. Some peptide families, such as defensins, are evolutionary conserved, while others are restricted to particular clades (e.g., thionins in plants).

In an era of rapidly developing microbial resistance to antibiotics, the search for new alternatives is of particular importance. Extremely varied in structure and created by nature itself, AMPs represent ideal scaffolds for the development of novel anti-infective agents. The main advantages of AMPs include their ability to act rapidly, affect a variety of pathogens and escape rapid resistance development [5]. Plant AMPs are synthesized on ribosomes. They usually consist of 20−50 amino acid residues, have a net positive charge, and are amphipathic, mostly hydrophilic, enriched in cysteine, glycine, and positively charged residues [6]. Based on the similarity in 3D structure and cysteine signature, plant AMPs are classified into several families, such as defensins, thionins, non-specific lipid transfer proteins (nsLTPs), α-hairpinins, hevein- and knottin-like peptides [7]. Despite obvious advantages, the practical use of natural AMPs is hampered by such drawbacks as limited quantities of peptides that can be isolated from natural sources, costly peptide synthesis, problems with appropriate folding, sensitivity to protease degradation, low activity at physiological salt concentrations, toxicity to human cells, etc. In this context, the main focus of research is on the development of synthetic analogues based on natural AMP sequences, devoid of limitations of natural AMPs. The design of AMP analogues includes truncation of sequences, mutation of specific amino acid residues, enrichment of the original sequence with certain types of residues, incorporation of unnatural amino acid residues, etc. [4].

Truncation of AMP sequences can provide highly active and simultaneously low-cost antimicrobials. This approach assumes knowledge of the sites of the AMP molecule associated with antimicrobial activity. Analysis of cysteine-rich peptides (CRPs) displaying antimicrobial activity showed that they all share a common structural element, the so-called γ-core, which is a conserved sequence pattern existing in three isoforms named dextromeric or D-isoform (NH_2_…[X_1–3_]-[GXC]-[X_3–9_]-[C]…COOH), levomeric 1 or L1-isoform (NH2…[C]-[X_3–9_]-[CXG]-[X_1–3_]…COOH) and levomeric 2 or L2-isoform (NH_2_…[C]-[X_3–9_]-[GXC]-[X_1–3_]…COOH) [8]. The γ-core adopts anti-parallel β-hairpin conformation with a central location in many antimicrobial peptides. It is positively charged and amphiphilic, which facilitates interactions with the membranes [8]. This motif was discovered in host defense peptides from phylogenetically distant organisms, suggesting that it plays an ancient and essential role in effector molecules governing host-pathogen interactions.

Since the discovery of the γ-core, a significant number of papers have appeared analyzing the role of this motif in the antimicrobial activity of AMPs and identifying amino acid residues essential for activity. The fact that γ-core determines the antimicrobial properties of the AMP was established by the studies in which the γ-core of one defensin was replaced by the γ-core of another, which was accompanied by a change in antimicrobial properties [9,10]. In subsequent studies, short peptides corresponding to the γ-core motifs were synthesized to assay their antimicrobial activity. Further “mutating” particular residues in these peptides enables the identification of the residues crucial for activity and even improves antimicrobial potency. Besides shedding light on the problem of structure-function relationship in plant AMPs, the γ-core-based short peptides can find applications in medicine and agriculture as new antimicrobial agents.

In this review, we summarized the available data on the γ-core peptides of plant AMPs. The material is grouped by botanical families of plants that served as a source of AMPs for the synthesis of γ-core peptides. We believe that the presented information will facilitate further studies of the antimicrobial determinants in CRPs and pave the way for the construction of novel short peptide antimicrobials on the basis of natural AMP sequences.

## 2. γ-Core Motifs of Defensins

**Figure 1:**
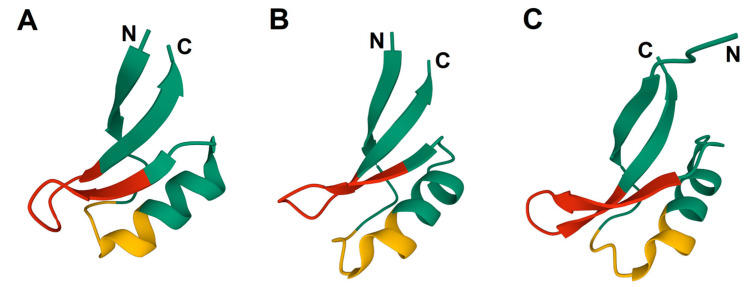
Solution structure of plant defensins: (**A**) MtDEF4 (PDB: 2LR3); (**B**) PvD1 (PDB: 6VPN); (**C**) HsAFP1 (PDB: 2N2Q). The γ-core motif is colored red. The α-core motif is colored yellow. The N- and C-termini are indicated by N and C, respectively.

Plant defensins belong to a highly conserved AMP family found in all multicellular organisms. They display a variety of functions, including antifungal, antibacterial, inhibition of proteases and amylases, inhibition of protein synthesis in cell-free systems, blockage of ion channels, heavy metal tolerance, and a role in sexual reproduction [11,12]. The molecular mechanisms underlying the biological activity of plant defensins are just beginning to become clear: they include interactions with specific membrane lipids of the pathogens, production of reactive oxygen species (ROS), and induction of cell wall stress. Despite sequence dissimilarity, plant defensins share a common 3D structure, which involves a triple-stranded antiparallel β-structure and an α-helix going in parallel to the β-sheet [13,14,15,16]. The γ-core represents a portion of the molecule encompassing the β2 and β3 strands and their connecting loop (Figure 1). Furthermore, a similar signature named α-core is located in the N-terminal half of the peptides, although it is not positively charged and does not adopt a β-hairpin conformation as the γ-core. Of plant AMPs, defensins were predominantly used in the γ-core structure/function studies.

### 2.1. Radish Defensins Rs-AFP1 and Rs-AFP2 (Brassicaceae)

Radish seed defensins Rs-AFP1 и Rs-AFP2 were among the first defensins isolated from plants [17], for which the problem of structure-function relationships was inferred. They inhibit the growth of filamentous fungi such as *Alternaria brassicola*, *Botrytis cinerea*, and *Fusarium culmorum* at micromolar concentrations, causing hyper branching of hyphae. The IC_50_ (concentration necessary for 50% inhibition of the pathogen growth) values for these fungi are as low as 2 µg/mL for Rs-AFP2 and vary from 5 to 15 µg/mL for Rs-AFP1 [17]. Radish defensins are active not only against plant pathogenic fungi but against human pathogens, including *Candida albicans*. Studies of the mode of action of Rs-AFP2 on *C. albicans* showed that the peptide interacted with the fungal glucosylceramides and induced apoptosis in the yeast cells. Further studies demonstrated that the defensin caused cell wall stress, mislocalization of septins, and accumulation of ceramides in *C. albicans* [18]. Moreover, both radish defensins prevented biofilm formation in *C. albicans* and acted synergistically with the antimycotics caspofungin and amphotericin B. Rs-AFP2 was more potent than Rs-AFP1 against planktonic and biofilm cultures [19]. Sequence analysis shows that the radish defensins possess the γ-core motif GSCNYVFPAHKC and the α-core motif GVCGNNNAC (Table 1, Figure S1).

The role of the γ- and α-core motifs in the antimicrobial activity of the radish defensins was explored in a number of studies. A series of 15-mer overlapping peptides covering the Rs-AFP2 sequence was produced by De Samblanx et al. (Table 1, Figure S1) [21]. Each peptide overlapped by 11 residues with the consecutive peptide. Peptide 4 covered the α-core motif, while peptide 9 encompassed the γ-core motif of Rs-AFP2. Antifungal activity assays against five plant pathogens (*F. culmorum*, *A. brassicola*, *Ascochyta pisi*, *B. cinerea,* and *Verticillium dahliae*) showed that only peptides 6, 7, 8, and 9 covering the region from Cys21 to Cys47 including the γ-core region were active against the selected fungi at MIC (minimal inhibitory concentration) from 30 to 250 µg/mL. *V. dahliae* was the most sensitive pathogen, equally potently inhibited by peptides 6−9 at a concentration of 30 µg/mL. Peptide 9 was also the most active against *F. culmorum*. Peptide 8 was the most active against *A. brassicola*. *A. pisi* and *B. cinerea* were poorly inhibited by all peptides. Of interest, peptide 10, covering most of the γ-core motif but devoid of the GXC signature, was inactive against the tested fungi. Conversely, the discovery of antifungal activity in peptides 6−8 lacking most of the γ-core points to the importance of the residues located N-terminally to the γ-core for antimicrobial activity. No correlation was observed between the peptide’s charge and antimicrobial activity: peptides 8, 9, and 10 had the same charge of +1. However, only peptides 8 and 9 were active against the tested fungi (Table 1).

A synthetic loop-mimetic peptide with a Cys36–Cys45 bond comprising most of the γ-core motif exhibited antifungal activity (Table 1). The Y38A substitution in this cyclic peptide decreased antifungal potency. The importance of Tyr38 for antifungal activity was further confirmed in antifungal assays of Rs-AFP2 substitution lines against *F. culmorum* [20]. Substitutions Y38G, F40M, P41∆, and K44Q in the γ-core motif reduced the antifungal potency of Rs-AFP2 (Table 1). The substitution of residues in close proximity to the GXCX_3-9_C signature, such as A31W and Y48I, also decreased inhibition. Interestingly, V39R substitution in Rs-AFP2 increased the antifungal potency compared to the native defensin (Table 1). Using Accelerated Molecular Dynamics, it was shown that the mutation V39R stabilizes the defensin structure and increases membrane deformation activity compared to the native peptide [24].

Another series of synthetic 19-mer peptides encompassing the γ-core region of Rs-AFP2 were synthesized, and their antimicrobial activity was assayed (Table 1) [22]. A peptide MBG01 covered residues from 31 to 49. In MBG02, the cysteine residues were substituted with α-aminobutyric acid. In MBG03, the cysteine residues were also substituted with α-aminobutyric acid, and the terminal residues Ala31 and Phe49 were substituted with cysteine. Peptide MBG04 was the same as MBG03. Only the α-aminobutyric acids at positions 36 and 45 were replaced by D-cysteines. By oxidation in DMSO, a disulfide bond was formed between the terminal cysteine residues in MBG03 and MBG04 and between the two D-cysteines in MBG04. Testing of the antifungal activity of the γ-core peptide MBG01 and its variants against *F. culmorum* showed that while Rs-AFP2 IC_50_ was 5 μg/mL, for MBG01, it was higher—33 μg/mL. Substitution of cysteines with α-aminobutyric acid preventing disulfide bond formation restored high antifungal activity of the peptide with IC_50_ of 8 μg/mL. The introduction of a disulfide bond between the terminal residues decreased the antifungal potency of the γ-core peptide MBG03 two-fold (IC_50_ = 17 μg/mL). The introduction of a second disulfide bond in MBG04 slightly enhanced the antifungal potency of the peptide (IC_50_ = 15 μg/mL). Thus, the highest activity compared to the original peptide MBG01 was observed in peptide MBG02, followed by MBG04 and MBG03, indicating that multimerization via disulfide bond formation is not necessary for the activity of the Rs-AFP2 γ-core peptides. Deletion of two amino acid residues (Ala31 and Arg32) from the N-terminus of MBG01 decreased the antifungal potency of the peptide MAT09 (IC_50_ = 41 μg/mL). Substitution of cysteine residues in peptide MAT09 with α-aminobutyric acid producing MBG05 abolished the antifungal activity of the peptide. Short, 6-mer peptides MBG06 and MBG08 were produced from the N-terminus of MBG01. Peptide MBG08 was identical to MBG06 but carried a substitution of cysteine with α-aminobutyric acid. The activity of the short MBG06 peptide was about five times lower than that of the original peptide MBG01. The peptide MBG08 was inactive. Similarly, 6-mer peptides MBG07 and MBG09 were produced from the C-terminal region of MBG01, and peptide MBG09 carried substitutions of cysteines with α-aminobutyric acid. Interestingly, while MBG09 was inactive, MBG07 was highly active (IC_50_ = 16 μg/mL). Thus, the activity of short MBG01-derived peptides depended on the presence of cysteine residues which possibly cause peptide dimerization or multimerization crucial for activity. Note that the peptide MAT02 harboring the α-core sequence was inactive against *F. culmorum* (Table 1).

In order to locate functionally important residues in the γ-core peptide MBG01, all the overlapping 13- to 20-mer peptides from position 26 to 49 in the Rs-AFP2 amino acid sequence were synthesized (Table 1) [22]. All cysteines were replaced by α-aminobutyric acid. The 20-mer peptide 30-49 containing the γ-core had the highest activity (IC_50_ = 5 μg/mL) identical to that of the native peptide Rs-AFP2. Deletion of Arg32 significantly decreased antimicrobial activity (IC_50_ = 247 μg/mL) [22]. Deletion of His33 further decreased antimicrobial potency (IC_50_ ˃ 400 μg/mL). A similar reduction in activity was observed when the C-terminal YF was deleted. Furthermore, it was also shown that the sequences less than 16 residues in length were, on the whole, less active than the longer peptides.

The antimicrobial properties of the γ-core of Rs-AFP2 against plant and human pathogens were further confirmed by Sathoff et al. (Table 1) [23]. The peptide appeared active against *Fusarium tricintcum* (IC_50_ = 5.3 μM). The bacteria *E. coli, Pseudomonas syringae* pv. *syringae* and *Sinorhizobium meliloti* were also sensitive to the Rs-AFP2 γ-core peptide. These results are in contrast with those of De Samblanx et al. [20], who showed the insensitivity of the γ-core-containing peptides of Rs-AFP2 to the bacterial pathogens in the concentration range from 3 to 400 μg/mL.

Thus, the antimicrobial properties of the γ-core-containing peptides of the radish defensins were shown in a number of studies. However, the accumulated data indicate that the antifungal activity of these defensins is not associated only with the γ-core motif regions. Analysis of different substitution lines of the radish defensins allowed De Samblanx et al. to conclude that there is a second antifungal site composed of the residues Thr10, Ser12, Leu28, and Phe49, which form a cluster in the 3D structure of the defensin molecule [20]. The residues of this cluster are not part of the α-core region of Rs-AFP2 either. The introduction of a positively charged residue within this cluster increases the antifungal activity of the peptide. Furthermore, analysis of the antifungal activity of Rs-AFP1 vs. Rs-AFP2 showed that Rs-AFP2 was 2−30 times more efficient in inhibiting spore germination of plant filamentous fungi than Rs-AFP1 [17], while amino acid comparison demonstrated that the peptides differ only by two amino acid residues (Glu5 in Rs-AFP1 is substituted for Gln in Rs-AFP2, and Asn27 in Rs-AFP1 is replaced by Arg in Rs-AFP2) in the N-terminal half of the molecule which increases the net positive charge of Rs-AFP2, but which are beyond the α- and γ-core regions.

### 2.2. Brassica Hybrid Defensins BhDef1 and BhDef2 (Brassicaceae)

From another Brassicaceae species found in Thailand, *Brassica hybrid* cv. Pule, two defensin genes named *BhDef1* and *BhDef2* were isolated [25]. The defensins BhDef1 and BhDef2 showed sequence similarity to defensins of other Brassicaceae species. BhDef1 displayed the highest sequence similarity (85% identity) with the γ-thionin from *Barbarea vulgaris*, and BhDef2, 78% similarity with the *Brassica napus* defensin. The γ-core motif of BhDef1 was identical to that of the radish Rs-AFP2 except for a single substitution of Asn for Tyr, while in BhDef2, the γ-core differed from the radish γ-core by six amino acid residues (Table 2, Figure S1). The α-core motif of BhDef1 was identical to that of Rs-AFP2.

Eight peptides were designed on the basis of BhDef1 and BhDef2 sequences (Table 2) [25]. Five peptides were derived from BhDef1. BhDef11 covered the first 15 amino acid residues. BhDef12 corresponded to the α-core of the defensin. BhDef12M was the same as BhDef12. Only three successive Asn residues were substituted for Arg to increase the positive charge (+3). BhDef13 encompassed the γ-core region with additional seven residues from the N-terminus, and finally, BhDef14 spanned the N-terminal half of the defensin molecule (26 residues) and had the highest net charge of +4. Three peptides originated from BhDef2. BhDef21 corresponded to residues 9−25 of the polypeptide chain. BhDef22 encompassed the γ-core region with additional seven residues from the N-terminus. BhDef23 included the α-core and was negatively charged (−1). The antifungal activity of the peptides was assayed against *Colletotrichum gloeosporoides* causing anthracnose disease (fruit rot). Antibacterial activity was assayed against *Staphylococcus aureus* (MRSA) and *Salmonella* typhi. BhDef12M showed an inhibition zone of 4 mm at a concentration of 512 µg/mL against *C. gloeosporoides*. BhDef13, BhDef14, and the negatively charged BhDef23 were also active but at a higher concentration of 1.024 mg/mL (inhibition zones of 7, 3, and 10 mm, respectively). Six peptides (BhDef11, BhDef12M, BhDef13, BhDef14, BhDef21, and BhDef23) were active against the Gram-positive bacterium MRSA and the Gram-negative bacterium *Salmonella* typhi. The MIC_99_ values varied from 0.93 to 2.93 mg/mL. BhDef14 covering the N-terminal half of the molecule was the most active peptide against both bacterial species, possibly due to the highest net positive charge of the peptide.

In summary, in contrast to the radish defensins, both the α-core and γ-core regions of *B. hybrid* defensins BhDef1 and BhDef2 were active against the fungus *C. gloeosporoides* and human pathogenic bacteria *Staphylococcus aureus* and *Salmonella* typhi, however, at high millimolar concentrations.

### 2.3. MsDef1 and MtDef4 (Fabaceae)

MsDef1 is an antifungal seed defensin of a perennial plant *Medicago sativa*, which inhibits the growth of filamentous fungi, including *F. graminearum* [9]. It belongs to morphogenic defensins inducing hyperbranching. MtDef4 is an apoplast-located defensin of *Medicago truncatula*, which is expressed in different tissues either constitutively or in response to stress factors. MtDef4 is a more potent inhibitor of *F. graminearum* than MsDef1 [9]. In contrast to MsDef1, MtDef4 is nonmorphogenic. Studies of the mode of action of these defensins against *F. graminearum* demonstrated that both MsDef1 and MtDef4 defensins induced plasma membrane permeabilization; however, MtDef4 was more efficient than MsDef1. In addition to fungi, MtDef4 was also active against Gram-negative and Gram-positive bacteria [23].

The γ-cores of both MsDef1 and MtDef4 defensins differ significantly in the amino acid sequences and the net charge (Table 3): +3 for MsDef1 and +6 for MtDef4. Both defensins possess a hydrophobic residue (Phe) in the γ-core region. Sagaram et al. studied structure-activity determinants in MsDef1 and MtDef4 defensins [9]. They showed that the replacement of the γ-core of MsDef1 for the γ-core of MtDef4 makes the MsDef1-γ4 variant almost as potent as MtDef4 against the fungus *F. graminearum* and abolishes hyperbranching characteristic of MsDef1. Different versions of the γ-core motifs of both defensins were synthesized (Table 3). Comparative analysis of the antimicrobial potency of MtDef4 γ-core variants against *F. graminearum* showed that (i) MtDef4 γ-core (GMA4) alone efficiently inhibited fungal growth, although the whole molecule was 3−4 times more active. (ii) The activity of the “extended” γ-core GMA4-C (with additional six residues from the C-terminus) was identical to that of the classical γ-core. (iii) The loop GMA4-L between the cysteines, which is a positively charged short peptide RGFRRR within the γ-core region, is also active, although to a lesser extent than the “classical” γ-core. (iiii) In the hexapeptide RGFRRR, Phe is important. Its substitution for Ala in GMA4-L1 dramatically decreases activity. (iiiii) Furthermore, the first Arg in this hexapeptide is also important. Its substitution for Ala in GMA4-L2 drastically decreases activity. It is worth noting that the sequence RGFRRR is found in defensins of different plant families (Poaceae, Amaranthaceae, Solanaceae, see below). Therefore, it is evolutionarily conserved.

Studies of the MsDef1 γ-core and its variants showed that (i) it was virtually inactive against *F. graminearum* (GMA1), although the whole molecule displayed rather potent antifungal activity. (ii) Addition of six residues to the C-terminus of the MsDef1 γ-core enhanced the antifungal activity of the MsDef1 fragment (GMA1-C) against *F. graminearum* compared to the classical γ-core. (iii) The loop GMA1-L between the two cysteine residues consisting of the sequence RDDFR, in contrast to the hexapeptide RGFRRR, was virtually inactive against *F. graminearum*.

Further studies of the role of the cationic loop RGFRRR in the mode of action of the MtDef4 defensin against *F. graminearum* showed that it is necessary for the defensin internalization into the cells of *F. graminearum* [28]. Replacement for AAAARR or RGFRAA destroys the ability of the MtDef4 defensin to enter fungal cells, demonstrating the importance of RGFR and RR sequences for this process. It was also shown that the loop RGFRRR interacts with high affinity with phosphatidic acid, a precursor of membrane phospholipids, and this interaction mediates the transport of MtDef4 into the fungal cell walls, possibly to further interact with intracellular targets.

The antifungal and antibacterial activity of the γ-core motifs of MsDef1 and MtDef4 (with six additional C-terminal residues) on other pathogens besides *F. graminearum*, was further studied by Sathoff et al. [23]. A special emphasis was made on the pathogens causing crown rot, which severely damaged *M. sativa*.

It was shown that the γ-core motif peptides of MtDef4 and MsDef1 displayed antifungal activity against crown rot pathogens in a micromolar range (Table 3). The γ-core motif of MtDef4 exhibited a wider spectrum of antifungal activity than MsDef1 γ-core. While the γ-core of MtDf4 was active against both *F. oxysporum* and *Phoma medicaginis*, the γ-core of MsDef1 was active only against *P. medicaginis* and inactive against *F. oxysporum* f. sp. *medicaginis* (at a concentration below 30 µg/mL) (Table 3). The antifungal activity of the γ-core from MtDef4 against *P. medicaginis* and *F. solani* was studied in more detail. It was demonstrated that this peptide suppresses spore germination, germ tube elongation, and mycelial growth. However, no morphological changes of spores or hyphae were observed.

Comparison of the activity of the full-length peptide MtDef4 with the activity of its γ-core showed that the intact defensin had higher activity against *P. medicaginis* and *F. oxysporum* f. sp. *medicaginis* than its γ-core (Table 3). Regarding the mode of action, the full-length defensin similar to the γ-core, induced inhibition of spore germination and hyphal growth.

In the same study, in addition to the antifungal activity, the antibacterial activity against plant and human pathogens of the γ-cores of the defensins MsDef1 and MtDef4 was also assayed [23]. The γ-core of MtDef4 displayed more potent antibacterial activity than the MsDef1 γ-core against *Pseudomonas syringae* pv. *syringae* but was less active against *Xanthomonas alfalfae* subsp. *alfalfae* (Table 3). Similarly to the antifungal activity, the activity of the full-length MtDef4 against *P. syringae* pv. *syringae* was much higher than that of its γ-core peptide. Against *X. alfalfae* subsp. *alfalfae*, the intact MtDef4 was also more potent than its γ-core.

Sathoff et al. also tested the activity of the γ-core of MtDef4 against four human bacterial pathogens: *Serratia marcescens*, *Enterobacter aerogenes*, *Enterococcus casseliflavus*, *Pseudomonas aeruginosa* [23]. The peptide was inactive at tested concentrations against *Enterococcus casseliflavus* and was highly active against *Enterobacter aerogenes* (IC_50_ = 2.3 µM) and *P. aeruginosa* (IC_50_ = 2.7 µM). Studies of the mode of action of MtDef4 γ-core against *Pseudomonas* species showed that MtDef4 γ-core damages bacterial OM leading to the production of pores [27]. It was also suggested that the MtDef4 γ-core antibacterial mode of action involves inhibition of translation.

The effect of MsDef1, MtDef4, and peptides derived from their γ-core motifs was also studied during colony initiation in the fungus *Neurospora crassa* [26]. It was shown that MsDef1 and MtDef4 and their peptides inhibited the germination of conidia and accompanying cell fusion in *N. crassa* with different efficiencies (Table 3). The hexapeptide RGFRRR derived from the γ-core motif of MtDef4 selectively inhibited cell fusion. It was demonstrated that MsDef1, MtDef4, and their peptides disturbed Ca^2+^ homeostasis, however, in distinct ways.

In addition to the γ-core motifs, MsDef1 and MtDef4 possess the α-core motifs (Table 3, Figure S1). In contrast to the γ-cores, they are not positively charged. Antifungal assays clearly demonstrated that the α-core motifs of both defensins did not exhibit antifungal activity against *F. graminearum* (IC_50_ > 48 µM) [9].

### 2.4. MtDef5 (Fabaceae)

MtDef5 is a highly cationic bi-domain defensin encoded by a single gene in the genome of the legume *M. truncatula*, which is predicted to be apoplast-located. It displays potent activity against a variety of filamentous fungi, being one of the most potent broad-spectrum antifungal defensins expressed in *M. truncatula*. It is 8 and 32 times more powerful growth inhibitor of *F. graminearum* than MtDef4 and MsDef1 [29]. Each domain of MtDef5 consists of 50 amino acid residues, which are connected by a 7-amino acid linker (Table 4). The net charge of MtDef5 is +16, and the net charge of its subunits is +7 for MtDef5A and +8 for MtDef5B. The γ-cores of the two subunits differ from each other by a single amino acid residue: Phe in MtDef5A is substituted with Ile in MtDef5B and are quite different from the γ-cores of MsDef1 and MtDef4 (Table 4, Figure S1). The antifungal activity of MtDef5 against *F. graminearum* is higher than that of individual subunits MtDef5A and MtDef5B (Table 4). Studies of the mode of action of this defensin showed that it induces membrane permeabilization and accumulation of ROS in both *F. graminearum* and *N. crassa* hyphae, which contributes, at least partially, to fungal growth arrest [29]. However, the internalization mechanisms of the defensin in *N. crassa* and *F. graminearum* are different. MtDef5 was shown to bind to several phospholipids in fungal membranes, mainly phosphatidylinositol monophosphates, and form oligomers in their presence, whereupon it enters the fungal cells and moves to the nucleus, intracellular membranes, and other subcellular compartments leading to their degradation. The role of different amino acid residues of the γ-cores in membrane permeabilization, lipid-binding, and antifungal activity was explored by substituting two adjacent residues in the γ-core of MtDef5 subunits with alanine [29]. The results showed that histidine and arginine residues present in the γ-core motif of each domain, His36, and Arg37 in MtDef5A and His93 and Arg94 in MtDef5B, are critical for oligomerization and antifungal activity of MtDef5; however, the antifungal activity of MtDef5 did not correlate with its ability to bind phospholipids.

The activity of MtDef5 defensin and the γ-core-containing region of MtDef5A was also studied against *M. sativa* crown rot pathogens (Table 4) [23]. MtDef5 was found active against *F. oxysporum* f. sp. *medicaginis* and *P. medicaginis* with IC_50_ values of 0.8 and 1.3 µM (for two *F. oxysporum* strains) and 1.5 and 1.6 µM (for two *P. medicaginis* strains). The γ-core containing peptide of MtDef5A was active only against *P. medicaginis* with IC_50_ values of 19.5 and 8.5 µM for two pathogen strains. Thus, the activity of the core peptide was lower than that of the full-length defensin. The γ-core of MtDef5A was also active against *F. solani* (IC_50_ = 4.1 µM).

The γ-core of MtDef5A, such as that of MtDef4, inhibited spore germination, germ tube elongation, and mycelial growth in *P. medicaginis* and *F. solani* without causing morphological changes of spores or hyphae [23].

In addition to the antifungal activity, MtDef5 and its subunits display antibacterial activity. It was shown that MtDef5 inhibits the growth of the plant bacterial pathogen—the Gram-negative bacterium *Xanthomonas campestris* but was ineffective against the Gram-positive *Clavibacter michiganensis* and *C. insidiosus,* possibly due to the inability of MtDef5 to cross the outer layer of peptidoglycan present in their cell walls [23,30]. The MtDef5B subunit exhibited more potent antibacterial activity than its parent bi-domain MtDef5 and MtDef5A subunit. MtDef5 and each of its two subunits trigger distinct morphological changes in *X. campestris* followed by bacterial cell death. They permeabilize the bacterial plasma membrane and translocate to the cytoplasm, bind to negatively charged DNA, and possibly kill bacterial cells by inhibiting DNA synthesis and/or transcription. The cationic amino acids (His36, Arg37, His96, and Arg97) present in the two γ-core motifs of MtDef5 that were shown to be essential for its antifungal activity are also important for its antibacterial activity (Table 4) [29].

Testing of the antibacterial activity of the γ-core of MtDef5A against the plant bacterial pathogens *X. alfalfae* subsp. *alfalfae* and *P. syringae* pv. *syringae* showed that the peptide was active against *P. syringae* pv. *syringae* (IC_50_ = 4.5 µM) and inactive against *X. alfalfae* subsp. *alfalfae* at a concentration of 30 µg/mL [23]. Note that the entire MtDef5 was also active only against *P. syringae* pv. *syringae* (IC_50_ = 0.1 µM) (Table 4).

Determination of the antibacterial activity of the γ-core of MtDef5A against four human bacterial pathogens: *S. marcescens, Enterobacter aerogenes, Enterococcus casseliflavus,* and *P. aeruginosa* showed that at tested concentrations, it was inactive only against *Enterococcus casseliflavus* and was highly active against *Enterobacter aerogenes* (IC_50_ = 2.8 µM) [23]. The activity against two other bacterial pathogens was also high (Table 4).

Studies of the mode of antibacterial action of MtDef5A γ-core-containing peptide on *P. aeruginosa* showed that in contrast to MtDef4 γ-core, MtDef5 γ-core did not cause considerable OM damage in the pathogen [27]. MtDef5 γ-core likely interacts with intracellular targets in sensitive pathogens.

### 2.5. PvD1 (Fabaceae)

Mello et al. studied the antifungal defensin PvD1 from *Phaseolus vulgare* seeds [31]. The defensin exhibited antifungal activity against yeasts (Table 5). Four peptides based on the γ-core sequence of PvD1 were synthesized: two peptides 15 amino acids long (γ_31-45_PvD1 and γ_31-45_PvD1^++^) and two peptides nine residues long (γ_33-41_PvD1 and γ_33-41_PvD1^++^) (Table 5, Figure S1). Both 15-mer peptides, in addition to the γ-core motif, contained two more residues from the N-terminus and four residues from the C-terminus. Note that PvD1 γ-core was identical to that of MsDef1. In all four peptides, cysteine residues were substituted with alanine to prevent the formation of disulfide bonds [31]. In one peptide of each pair, two negatively charged Asp residues were substituted with two Arg. The antifungal activity of the γ-core-containing peptides was assayed against two yeast species, *C. albicans,* and *C. buinensis*. The antifungal assays with the long peptides γ_31-45_PvD1 and γ_31-45_PvD1^++^ showed that yeast growth inhibition was considerably improved by substituting Asp with Arg, especially for *C. buinensis*. Complete inhibition was observed at a concentration of 73.4 µM for *C. albicans* and 18.35 µM for *C. buinensis*.

The antifungal assays with the short peptides γ_33-41_PvD1 and γ_33-41_PvD1^++^ demonstrated that inhibition of both yeasts by a non-modified peptide γ_33-41_PvD1 was poor [31]. The DD/RR substitution enhanced the degree of inhibition, especially for *C. buinensis*. Complete inhibition of *C. buinensis* was achieved at the γ_33-41_PvD1^++^ concentration of 36.7 µM. *C. albicans* was inhibited by all tested peptide concentrations. However, even at the highest concentration of 293.6 µM, only 63% inhibition was reached.

The mode of action of the short peptide γ_33-41_PvD1^++^ was further studied against a more sensitive yeast species *C. buinensis* [31]. It was shown that *C. buinensis* cells lost their viability in the presence of the peptide. Treatment of *C. buinensis* cells with γ_33-41_PvD1^++^ at a concentration of 73.4 µM resulted in a 100% loss of viability, demonstrating that the peptide exerted a fungicidal effect on this pathogen.

At a concentration below MIC (25 µM), γ_33-41_PvD1^++^ caused membrane permeabilization [31]. The peptide also induced oxidative stress manifested by ROS production, dysfunction of mitochondria, and activation of metacaspases. It was suggested that yeast killing occurred due to programmed cell death via an apoptotic pathway.

Thus, the authors concluded that (i) the longer peptides extending beyond the γ-core motif of PvD1 have higher antifungal activity than the shorter ones. This observation means that not only the γ-core motif is responsible for the antifungal properties of defensins, but the surrounding residues are also involved. (ii) Increasing the net positive charge of the γ-core-containing peptides enhances their antifungal potency.

### 2.6. VuDef1 (Fabaceae)

Toledo et al. studied the activity of the VuDef1 defensin from cowpea *Vigna unguiculata* [32]. This defensin, in combination with an LTP, displayed inhibitory activity against filamentous fungi, *Leishmania amazonensis,* and insect α-amylases [33,34,35]. A synthetic peptide A_36,42,44_γ_32–46_VuDef named DD corresponding to the γ-core of this defensin with three cysteines replaced with alanine, and its variants were produced (Table 6, Figure S1) [32]. Note that the γ-core of VuDef1 was identical to the PvD1 γ-core except for the Val/Phe substitution. In order to increase the positive charge of the γ-core peptide, in one variant A_36,42,44_R_37,38_γ_32–46_VuDef named RR, two Asp residues were substituted with two Arg residues. In the second variant D-A_36,42,44_R_37,38_γ_32–46_VuDef named D-RR, the sequence was the same as in RR, but all amino acids were D-enantiomers to reduce protease degradation and mammalian cell toxicity. In the third variant, designated A_42,44_R_37,38_W_36,39_γ_32–46_VuDef and named WR, tryptophan residues instead of Ala36 and Val39 residues were introduced (the numbering of amino acid residues is given as in the original article [32]). Tryptophan residues in AMPs interact with high efficiency with membranes of pathogenic microorganisms, and their incorporation into the designed AMP sequence increases its antimicrobial potency [36]. A synthetic peptide DD showed activity on the parasite *L. amazonensis* similar to that of Vu-Def (Table 6). The interaction between DD and *L. amazonensis* resulted in parasite inhibition by the activation of an apoptotic-like cell death pathway [37]. The γ-core-based peptides were also assayed against five *Candida* species (*C. albicans, C. buinensis, C. parapsilosis, C. tropicalis* and *C. pelliculosa*) and *S. cerevisiae* (Table 6) [32]. The DD peptide showed no activity against yeasts. In contrast, RR peptide at a concentration of 18.5 µM inhibited the growth of *C. albicans, C. buinensis*, and *C. tropicalis* by 47.5, 100, and 72.1%, respectively; however, *S. cerevisiae*, *C. parapsilosis,* and *C. pelliculosa* were insensitive to the peptide. D-RR at a concentration of 18.5 µM inhibited the same *Candida* species as RR, however, with greater efficiency. The authors suggest that higher potency of D-RR could be associated with higher resistance to protease degradation [32]. The peptide WR, which was the most hydrophobic and cationic of all tested peptides, had the highest activity on the sensitive yeast species. It inhibited the growth of *S. cerevisiae, C. albicans, C. buinensis,* and *C. tropicalis* by 26.1, 96.2, 98.5, and 58.2%, respectively, although it was unable to suppress the growth of *C. parapsilosis* and *C. pelliculosa*. The MIC values for inhibition of *C. albicans* by D-RR peptide and WR were 23 µM and 18.5 µM, respectively. Thus, the designed peptides were more potent against the sensitive yeast species than the original DD peptide.

The peptides displayed fungicidal activity on the tested yeasts [32]. The lethal dose (LD_100_) values for *C. albicans* were 36.5 μM for D-RR and 27.5 μM for WR. All peptides, especially WR, were found non-toxic for mammalian cells (human monocytes and murine macrophages) at concentrations below 50 µM.

### 2.7. DefSm2-D (Asteraceae)

A cDNA was cloned from *Silybum marianum* flower buds. It encoded a putative antimicrobial protein DefSm2 of 95 amino acids containing an N-terminal defensin domain (DefSm2-D) and a C-terminal Arg-rich and Lys-rich domain [38]. The defensin DefSm2-D showed 84% sequence identity with the antifungal defensin DmAMP1 from *Dahlia merckii* seeds, which inhibited the growth of *F. solani*, *F. culmorum*, *N. crassa,* and *S. cerevisiae* [39]. The γ-core motif of DefSm2-D is identical to that of DmAMP1. Four short peptides originating from the γ- and α-core motifs of DefSm2-D were synthesized (Table 7) [38]. SmAP_α1-21_ included the N-terminal region of the defensin molecule, and the α-core motif followed by glycine. SmAP_α10-21_ was a shorter peptide containing only the α-motif with two additional residues from the N-terminus and one glycine from the C-terminus. SmAP_γ27-44_ contained the γ-core motif with six additional residues from the N-terminus, while SmAP_γ29-35_ was the shortest peptide of all, containing only GAC from the γ-core motif and four additional residues from the N-terminus. CD spectra showed that the peptides were predominantly in a random coil conformation.

Peptide SmAP_γ29-35_ at concentrations up to 100 µM did not show any activity against *F. graminearum*. Three other peptides were active: the activity of the longer peptide was higher than that of the shorter one. MIC values were 32 µM for SmAP_α1-21_, 70 µM for SmAP_α10-21,_ and 20 µM for SmAP_γ27-44_. All three active peptides contained a tryptophan residue which plays a crucial role in the interactions with membranes, anchoring the peptide at the membrane interface [36].

Studies of the mode of action of the active peptides showed that all three peptides induced membrane permeabilization of *F. graminearum* conidia as judged by propidium iodide uptake. In the presence of the α-core-derived peptides macroconidia aggregated into network-like clusters. Clusters were not formed with the SmAP_γ27-44_ peptide. Transmission electron microscopy showed that SmAP_α1-21_, in contrast to SmAP_γ27-44_, induced granulation of the cytoplasm. Both in the presence of SmAP_α1-21_ and SmAP_γ27-44_, the appearance of a large number of peroxisomes was recorded. The results obtained indicated that the mode of action of α-core-derived peptides is different from that of γ-core-derived peptides.

### 2.8. Atr-DEF2 (Amaranthaceae)

Moyer et al. studied the mode of antibacterial action of the γ-core motif peptide from the predicted defensin Atr-DEF2 of *Amaranthus tricolor* [40]. In Atr-DEF2, the γ-core motif is identical to that of MtDef4 (Figure S1). The peptide Atr-DEF2(G39-C54) spanning the γ-core and the C-terminal residues except for the C-terminal Ala of Atr-DEF2 was synthesized (Table 7), and its activity against the Gram-negative human pathogenic bacteria *Escherichia coli* and *Klebsiella pneumoniae* was assayed [40]. The γ-core motif peptide was much more active against *E. coli* (IC_50_ = 9 μM) than against *K. pneumonia* (IC_50_ = 68 μM). The proteomic changes in *E. coli* induced by Atr-DEF2(G39-C54) were explored using label-free quantitative proteomics, and it was discovered that of the 1598 proteins, the abundance of 51 proteins decreased, and the abundance of 82 proteins increased. It was found that six proteins involved in the lipid A aminoarabinose modification pathway displayed increased abundance in Atr-DEF2(G39-C54)-treated *E. coli* cells. This suggests that *E. coli* was up-regulating signaling related to OM stress response. Furthermore, proteomics analysis demonstrated that the peptide Atr-DEF2(G39-C54) triggered iron deficiency. In vitro assays proved that it reduced the concentration of aqueous Fe^3+^ and chelated Fe^2+^. Accordingly, Atr-DEF2(G39-C54) influenced the composition of the OM and iron homeostasis in the pathogen, which reacted to OM and iron deficiency stress.

### 2.9. Spinach So-D2 (Amaranthaceae)

Seven defensins So-D1−7 were isolated from *Spinacia oleraceae* leaves [41]. The So-D2 was completely sequenced. Antimicrobial assays showed that So-D2 was highly active against plant pathogenic bacteria and fungi: *C. michiganensis*, *Ralstonia solanacearum*, *F. culmorum,* and *F. solani* [41]. The defensin was also shown to be highly active against human pathogens, including multidrug-resistant (MDR) strains of *P. aeruginosa* and *C. albicans* [42]. It also inhibited MDR strains of *E. coli* and *Klebsiella pneumonia*, although less effectively. The So-D2 was shown to inhibit biofilm formation in MDR *P. aeruginosa* and *C. albicans*. Studies of the mode of action of the defensin demonstrated that it disturbed the integrity of the cell membranes of *P. aeruginosa* and *C. albicans* [42].

The modified γ-core motif peptide of So-D2 defensin with an extension of six amino acid residues and Cys/Leu substitution at the C-terminus was synthesized and assayed against the fungal and bacterial alfalfa crown rot pathogens (Table 7) [23]. The peptide was found active against *F. oxysporum* f. sp. *medicaginis* with IC_50_ of 33.1 μM and highly active against two *P. medicaginis* strains with IC_50_ values of 6.4 and 6.1 μM (Table 7). The peptide also efficiently suppressed the growth of *F. solani* (IC_50_ = 13.8 μM). The So-D2 γ-core peptide inhibited the growth of the bacterial pathogens *X. alfalfae* subsp. *alfalfae* (IC_50_ = 19.3 μM) and *P. syringae* pv. *syringae* (IC_50_ = 25.9 μM), although the γ-core peptide of MtDef4 was more potent (see above).

### 2.10. BcDef (Solanaceae)

A defensin gene *BcDef* was cloned from *Brugmansia* x *candida*, a medicinal ornamental plant [43]. The predicted BcDef defensin shares high sequence similarity (78−85%) with Solanaceae class 1 defensins. The BcDef defensin has both the γ- and α-core motifs. The γ-core-containing peptide BcDef1 consisting of 17 amino acid residues (net charge of +4) of this defensin was synthesized (Table 7) [43]. The peptide displayed inhibitory activity against Gram-positive and Gram-negative bacteria with MIC values from 15 to more than 251 µM (Table 7). The highest activity (MIC = 15.7 µM) was observed against *Staphylococcus epidermidis*. Moderate activity (MIC = 31.4 µM) was recorded against *S. typhimurium*. The activity against other pathogens (*Vibrio cholera*, *Shigella sonnei*, *E. coli*, *Enterococcus faecalis*, *Bacillus cereus*, *S. aureus*) was lower (Table 7). It was shown that BcDef1 kills *S. epidermidis* cells by triggering membrane and cell wall damage, including alteration of the membrane potential and permeability without cytotoxicity to normal mammalian cells [43]. In addition to antimicrobial properties, the peptide displayed antioxidant activity.

### 2.11. Tomato SolyC07g007760 (Solanaceae)

A very similar to the γ-core peptide BcDef1, a 17-mer peptide named SolyC, was synthesized by Rigano et al. [44]. It encompassed the γ-core region of a tomato defensin SolyC07g007760 (Table 8, Figure S1). The antibacterial activity of SolyC against important human pathogens was assayed [44]. At a low peptide concentration, SolyC showed antimicrobial activity against the Gram-negative bacteria *Salmonella enterica* serovar Paratyphi, *E. coli*, and *Helicobacter pylori* (MIC = 15 µg/mL). At a higher concentration, it also inhibited the growth of the Gram-positive bacteria *S. aureus*, *S. epidermidis,* and *Listeria monocytogenes* (MIC = 40 µg/mL). Meanwhile, SolyC displayed very low antibacterial activity against the probiotic bacteria *Lactobacillum plantarum* and *L. paracasei*.

The peptide showed very low (< 5%) hemolytic activity and had no cytotoxic effects at concentrations from 60 to 120 µg/mL against THP-1 human acute monocytic leukemia cells [33]. In addition, the SolyC peptide displayed anti-inflammatory activity in vitro by down-regulating the level of the proinflammatory cytokines TNF-α and IFN-γ. Thus, for this γ-core peptide, a dual function as an antimicrobial and anti-inflammatory agent was demonstrated.

In the following study, several derivatives of SolyC were produced, and their antibacterial activity against the Gram-positive (*S. aureus, S. epidermidis*, *L. monocytogenes*) and Gram-negative bacteria (*H. pylori* and *S. enterica*) was studied [45]. These peptides included: a linear SolyC, a truncated linear SolyC-t, lacking three amino acids from the N-terminus, SolyC1, in which the third Cys was replaced by Ser, a truncated SolyC1-t, SolyC2, in which Cys2 was replaced by Ser, truncated SolyC2-t, and oxidized forms of these peptides—SolyC1-ox, SolyC1-t-ox, SolyC2-ox, and SolyC2-t-ox, with a single disulfide bond each. The antimicrobial activity (MIC_100_) of all the peptides was in the range from 10 to 100 µg/mL (Table 8).

It was shown that, in general, the peptides were more active against the Gram-negative bacteria than against Gram-positive bacteria (Table 8) [45]. Linear peptides were more active than their truncated forms. For the intact SolyC, truncation resulted in the reduction in activity against the Gram-positive *Staphylococcus* spp. However, no differences in activity against the Gram-negative bacteria were observed (Table 8). Against *L. monocytogenes*, all modified peptides had the same antimicrobial activity (MIC_100_ = 80 µg/mL) as SolyC. Linear peptides with two cysteines, SolyC1 and SolyC2, showed reduced activity compared to SolyC against the Gram-positive *Staphylococcus* spp. All oxidized peptides were highly active against the Gram-negative bacteria (MIC_100_ = 15−20 µg/mL). The linear peptide SolyC2 displayed the same antimicrobial activity against the Gram-negative bacteria as the original peptide SolyC, whereas the linear SolyC1 was less active. The activity of all modified peptides against the Gram-positive bacteria was, in all cases, lower than that of SolyC. The authors suggest that the interaction of the peptides with the membranes of Gram-negative bacteria is stronger when the positive charges are exposed, which is observed when the peptides are in the oxidized form. Thus, the formation of an intramolecular disulfide bond stabilized the peptides in the “active” conformation.

It was also demonstrated that all peptides up to a concentration of 90 µg/mL showed low hemolytic activity (below 30%) [44]. It was also shown that the peptide SolyC2 with high selectivity interacted with the bacterial OM.

### 2.12. Tomato SlDEFL2 and SlDEFL4 (Solanaceae)

In tomato transcriptomes, two defensin genes, *SlDEFL1* and *SlDEFL2*, which were upregulated by *F. oxysporum* infection and fungal resistance inducers, were discovered [47]. They encoded two peptides, SlDEFL2 and SlDEFL4, which harbored nearly identical γ-core motifs [46]. Two γ-core-containing peptides named γ_58-74_SlDEFL2 and γ_58-74_SlDEFL4, respectively, were synthesized (Table 8, Figure S1). They differed by two amino acid residues at positions 2 (Ser/Thr) and 5 (Asp/Asn), resulting in a higher positive charge of γ_58-74_SlDEFL4 (+5 vs. +4 for _γ58-74_SlDEFL2). Note that DEFL-derived peptides were highly similar to SolyC. Antimicrobial activity of these two γ-core peptides was assayed against a panel of nine pathogens, including yeasts *Cryptococcus neoformans* and *Candida albicans* affecting immunocompromised individuals, plant pathogenic bacteria *Clavibacter michiganensis*, *Pseudomonas savastanoi* and *Pectobacterium carotovorum*, four *Fusarium* species, *Bipolaris sorokiniana* and *Botrytis cinerea* [46]. Both γ-core peptides were found to be highly active against yeasts, especially *C. neoformans* (Table 8). Both Gram-positive (*C. michiganensis*) and Gram-negative bacteria (*P. savastanoi* and *P. carotovorum*) were sensitive to the peptides. The IC_50_ for inhibition of *C. michiganensis* was 19.8 and 21.5 µM for γ_58-74_SlDEFL2 and γ_58-74_SlDEFL4, respectively. At the highest tested concentration of 300 µM, a positive correlation between the net charge and inhibitory activity against bacteria was observed: γ_58-74_SlDEFL4 was more efficient than γ_58-74_SlDEFL2. For γ_58-74_SlDEFL4, the degree of inhibition varied from 100% against *P. savastanoi* to 81% against *P. carotovorum*, while γ_58-74_SlDEFL2 was less active against all tested bacteria. In contrast to bacteria and yeasts, the activity of both γ-core peptides against fungal pathogens was similar. *F. verticillioides* and *F. solani* were insensitive to γ_58-74_SlDEFL2 and γ_58-74_SlDEFL4 at all tested concentrations, while IC_50_ for inhibition of *F. culmorum* was 44.8 and 42.3 µM and IC_50_ for inhibition of *F. oxysporum* was 165.8 and 124.8 µM, respectively. *B sorokiniana* appeared to be insensitive to γ_58-74_SlDEFL2 and γ_58-74_SlDEFL4; conversely, *B. cinerea* was inhibited by 31–45% at the highest tested concentration. Studies of the mode of action of the highly active peptide γ_58-74_SlDEFL4 on *C. albicans* cells using staining with propidium iodide showed that the peptide induced membrane permeabilization suggesting that membrane disruption might be the cause (or one of the causes) of the pathogen killing [46].

### 2.13. HsAFP1 (Saxifragaceae)

HsAFP1 is a defensin from seeds of coral bells *Heuchera sanguine*, which is active against plant pathogenic fungi, such as *B. cinerea*, *Verticillium albo-atrum,* and *F. culmorum* [39]. It is also active against *Saccharomyces cerevisiae* and *C. albicans*, inducing apoptosis and preventing biofilm formation in *C. albicans*. HsAFP1 acts synergistically with caspofungin and amphotericin B against *C. albicans* planktonic cells and biofilms [48]. Six 24-mer synthetic peptides with 18-mer overlap spanning the entire HsAFP1 sequence were synthesized, and their activity towards *F. culmorum* and *C. albicans* planktonic cultures and biofilms was studied (Table 9) [48]. Cysteine residues were replaced by α-aminobutyric acid. Peptides 5 and 6 encompassed the γ-core signature. Peptide 5 included the γ-core and 12 residues from the N-terminus of the γ-core, while peptide 6 covered the γ-core sequence with six additional residues from the N- and C-termini. Peptides 1−3 harbored the α-core motif. None of the linear fragments inhibited the growth of *F. culmorum* up to the highest tested concentration of 1.5 μM, while the recombinant HsAFP1 inhibited the growth of this pathogen with IC_50_ = 0.45 μM. Furthermore, the synthetic peptides did not suppress the growth of *C. albicans* in contrast to the full-length defensin. Only HsLin06 inhibited *C. albicans* biofilm formation to the same extent as the recombinant HsAFP1 (Table 9). Thus, the importance of the γ-core and its adjacent regions for antibiofilm activity was demonstrated. HsLin03 and HsLin05 also inhibited *C. albicans* biofilm formation. However, their efficiency was much lower than that of HsLin06 (10 or 15 times lower). Other linear peptides were inactive against *C. albicans* biofilms up to a concentration of 175 μM. Only three linear peptides, HsLin06, HsLin01, and HsLin05, acted synergistically with caspofungin in preventing biofilm formation [48].

### 2.14. Rice OsAFP1 (Poaceae)

The rice OsAFP1 defensin is highly active against the plant pathogenic fungi *Pyricularia oryzae*, *Rhizoctonia solani,* and *Gibberella fujikuroi*, but is inactive against bacteria, such as *Burkhalderia plantarii*, *B. glumae,* and *Acidovorax avenae* (Table 10) [49]. Eight 10-mer overlapping peptides covering the entire defensin sequence were synthesized (Table 10) [49]. Peptide 7 covered the γ-core motif without the GXC submotif. Peptide 8 encompassed the C-terminal part of the γ-core motif plus the C-terminal region of the defensin molecule. Peptides 1 and 2 from the N-terminal region and peptides 7 and 8 from the C-terminal region possessed antifungal activity against *P. oryzae.* The N-terminal peptide 1 had the highest activity (IC_50_ = 0.41 µg/mL), which was higher than that of the intact defensin (IC_50_ = 0.99 µg/mL). Peptides 2, 7, and 8 had approximately the same activity as the intact defensin (Table 10). Note that peptide 3, encompassing the α-core motif, was inactive. The obtained results indicate that activity against *P. oryzae* depends both on the N-terminal and the γ-core containing regions of the OsAFP1 molecule.

In addition to plant pathogenic fungi, rice OsAFP1 was found to be fungicidal to *C. albicans* cells (Table 10) [50]. Studies of the mode of action of OsAFP1 showed that it induces apoptosis 0.5 h after treatment. The defensin was also active against *Saccharomyces cerevisiae*. However, this defensin was inactive against human pathogenic bacteria, such as *E. coli*, *Porphyromonas gingivalis*, *Streptococcus mutans*, *Staphylococcus aureus*, and *Propionibacterium acnes*. The same eight overlapping peptides of OsAFP1, as were tested against plant pathogenic fungi, were assayed against *C. albicans* (Table 10). Peptides 1, 2, 7, and 8 possessed antifungal activity with IC_50_ = 6, 19, 10, and 13 μM, respectively. The highest activity was displayed by peptides 1 and 7. Again peptide 3 encompassing the α-core motif was inactive. Thus, the N-terminal and the γ-core containing regions of the OsAFP1 molecule are important for the antifungal activity. To identify the residues essential for antifungal activity, in the peptide 7 region of OsAFP1, six amino acid residues with voluminous side chains Lys35, His37, Leu39, Glu40, Arg41, and Lys42 were successively replaced by alanine [50]. The activities of L39A and R41A mutants were very low, indicating the vital role of these residues in antifungal activity (Table 10). Thus, the γ-core motif, especially Leu39 and Arg41, plays an important role in OsAFP1 antifungal activity.

### 2.15. Maize ZmESs (Poaceae)

Four genes named *ZmES1−4* (*Zea mays* embryo sac) were isolated from the cDNA library of maize egg cells [51]. They code four highly similar defensins with sequence identity from 91% to 97%. Studies of the biological activity of ZmES1−4 peptides showed that ZmES4 induced pollen tube burst by opening the potassium channel KZM1. ZmES1−4 peptides also inhibited the germination of *F. graminearum* conidia and *Ustilago maydis* spores (Table 11) [52].

To reveal regions essential for pollen tube burst and antifungal activity of the ES peptides, five peptides (13–16 amino acids in length) named ES-a, ES-b, ES-c, ES-d, and ES-e, which covered the entire sequence of ES4, were synthesized (Table 11) [52]. Studies of the effect of ES4 and ES4-derived peptides on pollen tube burst in maize showed that the ES-d peptide encompassing the γ-core was even more active than the full-length peptide; at a concentration of 0.5 µM, the induction of pollen tube burst amounted to 82.5%. Peptides derived from other ES regions were unable to induce pollen tube burst. In order to identify amino acid residues involved in maize pollen tube burst, ES-d, and its 15 mutated derivatives were applied to germinated maize pollen. The results showed that mutations of Leu3, Ile4, or Tyr15 in ES-d inhibited pollen tube burst to less than 8%, thus pointing to the crucial role of these residues in this process. Studies of the antimicrobial effect of ES-c and ES-d on two maize pathogens, *F. graminearum,* and *U. maydis,* demonstrated that at high peptide concentrations (10–90 µM), both peptides displayed inhibitory activity, while other peptides did not. Mutations of Gly8, Tyr9, or Thr10 in ES-c; and mutations of Lys13, Cys14, or Tyr15 in ES-d significantly increased fungal germination. Thus, the γ-core region of ES4 and its neighboring residues from the N-terminus sequence is important for the antifungal activity. The mode of action of ES-d and ES-c peptides was shown to be different for different pathogens [52]. The ES-d bound only to the cell surfaces of *F. graminearum*, while the ES-c peptide bound to cell surfaces and accumulated inside the cells. In *U. maydis*, both peptides accumulated inside the cells.

### 2.16. Wheat TkDEFLs (Poaceae)

Eight γ-core motif peptides of the defensins TkDEFL1-11, 1-12, 1-16, 1-20, 1-23, 1-32, 1-36, and 1-40, which were predicted in the wheat *Triticum kiharae* during RNA-seq data analysis, were synthesized and their antimicrobial properties were explored [53]. Several γ-core peptides showed high sequence similarity (Table 12, Figure S1). The TkDEFL1-16 γ-core peptide possessed a highly basic hexapeptide RGFRRR, which was discovered in a number of defensins, including MtDef4, SolyC07g007760, SlDEFL2, and SlDEFL4. Four synthetic γ-core peptides were designed on the basis of the sequences of 4-Cys-containing defensins TkDEFL4-4, 4-8, 4-20, and 4-37. In addition to the full-length TkDEFL4 γ-core peptides, their shortened versions were also synthesized. These truncated variants represented the loops between the second and the third cysteine residues with or without the adjacent cysteine residues (Table 12). HvDEFL4-1_67-77_, corresponding to the loop between the second and the third cysteines in barley HvDEFL4-1, was also synthesized.

The synthetic peptides were assayed against nine pathogens, including the yeasts affecting humans, plant pathogenic bacteria, and fungi: *Candida albicans*, *Cryptococcus neoformans*, *Clavibacter michiganensis*, *Pseudomonas savastanoi, Pectobacterium carotovorum*, and four *Fusarium* species (*F. oxysporum*, *F. culmorum*, *F. solani,* and *F. verticillioides*) [53]. All tested peptides displayed antimicrobial properties (Table 12). As expected, their activity varied depending on the pathogen. Most γ-cores of classical defensins displayed high antimicrobial activity against the vast majority of the pathogens tested. The most active peptide was TkDEFL1-16_65-82._ The IC_50_ values for inhibition of the yeasts by this peptide were 4.4 µM and 14.6 µM for *C. neoformans* and *C. albicans*, respectively. IC_50_ for inhibition of the Gram-positive bacterium *C. michiganensis* was 14.6 µM, 12.1 µM, and 20.7 µM for the fungi *F. oxysporum* and *F. culmorum*, respectively. The high antimicrobial potency of TkDEFL1-16_65-82_ might be associated with its highest net charge at neutral pH (+5) compared to other peptides. The least active γ-core peptide designed from the sequences of the classical defensins was the peptide TkDEFL1-11_55-68_. Remarkably, one of the most active peptides, TkDEFL1-32_55-68_ differed from the least active TkDEFL1-11_55-68_ by two amino acid residues (IS in TkDEFL1-11_55-68_ is replaced by FR in TkDEFL1-32_55-68_), which increases the net charge of TkDEFL1-32_55-68_. Thus, FR is vital for the antimicrobial potency of TkDEFL1-32_55-68_. Comparison of the antimicrobial properties of two γ-core motif peptides TkDEFL1-23_65-82_ and TkDEFL1-40_65-82_, which differ by a single residue (Y13F) between the second and the third cysteines showed that they are very similar pointing to the minor role of this substitution for antimicrobial activity.

Studies of the antimicrobial activity of seven peptide fragments of 4-Cys-containing defensins showed that all of them suppressed the growth of yeasts, however, the efficiency of inhibition varied, and usually, the activity of the longer peptides was higher than that of the truncated variants [53]. For example, TkDEFL4-20_86-110_ was more potent than TkDEFL4-20_92-102_, and TkDEFL4-37_90-102_ was more efficient than TkDEFL4-37_91-101_ in suppressing the growth of *C. neoformans* (Table 12). TkDEFL4-37_90-102_ was also more effective in inhibiting the growth of *P. savastanoi* and *P. carotovorum*. Furthermore, the peptide TkDEFL4-20_86-110_ exhibited the highest activity of all peptides against *C. michiganensis* at 300 µM (91% inhibition), and TkDEFL4-37_90-102_ was the most effective against *P. savastanoi*. We can hypothesize that for the inhibition of these pathogens, oligomerization of the peptides via disulfide bond formation is required. Against *F. culmorum,* the highest activity (60% inhibition at 150 µM) was displayed by TkDEFL4-20_86-110_. The same peptide was the most potent against *F. oxysporum* (54% inhibition at 150 µM). In contrast to the activity against yeasts and bacteria, no positive correlation was observed between the length of the peptide fragment and its activity against all *Fusarium* fungi.

The mode of action of the γ-core peptides TkDEFL1-16_65-82_, TkDEFL1-32_55-68_, TkDEFL4-20_86-110_, TkDEFL4-37_90-102_ and HvDEFL4-1_67-77_ on *C. albicans* and *C. neoformans* cells was studied by fluorescent microscopy. The results clearly demonstrated that all tested peptides induced membrane permeabilization leading to the accumulation of the fluorescent dye inside the yeast cells.

### 2.17. Olive tree OefDef1.1 (Oleaceae)

OefDef1.1 is a highly cationic His- and Tyr-rich *Olea europaea* defensin with a high percentage of hydrophobic residues, which belongs to the Oleaceae-specific defensin subfamily [10]. OefDef1.1 inhibits the growth of *B. cinerea*, *F. graminearum*, *F. oxysporum,* and *F. virguliforme* (Table 13). Surface application of the peptide on *Nicotiana benthamiana* and lettuce leaves, followed by inoculation with *B. cinerea* conidia, resulted in reduced gray mold symptoms. Studies of the mode of action of OefDef1.1 showed that the peptide rapidly permeabilized the plasma membrane of the conidia and germlings of *B. cinerea*. The latter were more sensitive to the peptide’s action.

The γ-core of OefDef1.1 is quite different from those of other defensins (Table 13, Figure S1) [10]. In order to elucidate its role in the antifungal activity of OefDef1.1, two OefDef1.1 variants were generated: in one, named OefDef1.1_V1, the γ-core motif was replaced by that of MtDef4, in another OefDef1.1_V2 variant, by the core motif of DmAMP1 from *Dahlia merckii* (Table 13). Note that the sequence of OefDef1.1 has a certain sequence similarity with DmAMP1. However, the sequence of the γ-core is different. The antifungal activity of both peptide variants against *B. cinerea* and *F. oxysporum* was assayed. The variant OefDef1.1_V1 with the γ-core of MtDef4 lost 50% activity compared to the wild-type peptide (Table 13). In contrast, the activity of the second variant was slightly higher than that of the wild-type OefDef1.1. Although the variant OefDef1.1_V1 was less active in vitro than the wild-type peptide, it was the most effective in reducing lesions caused by *B. cinerea* on detached *N. benthamiana* and lettuce leaves, namely, the peptide with a higher net charge was more effective than the peptide with a lower charge. The other conclusion was that in vitro antifungal activity does not always correlate with in planta activity.

In order to identify the residues important for the antifungal activity, alanine scanning mutagenesis of the sequence between the two cysteines in the γ-core motif of OefDef1.1 was performed [10]. Three variants designated OefDef1.1_V3 (with three neighboring N-terminal amino acid residues replaced with alanine), OefDef1.1_V4 (with the next three residues replaced with alanine), and OefDef1.1_V5 (with four C-terminal residues replaced with alanine) were obtained (Table 13). None of the variants lost in vitro antifungal activity. The activity of OefDef1.1 variants was similar to that of the wild-type peptide. Thus, specific amino acids within the γ-core motif are not critical for the antifungal activity of OefDef1.1. Hydrophobicity rather than the net charge may be a more significant contributor to the antifungal activity of this peptide.

## 3. Non-Defensin γ-Cores

In addition to the γ-core motif peptides of defensins, the γ-core motif peptides designed from the CRPs belonging to non-defensin families were also studied [46,53]. Three peptides, γ_48-65_SlSN2, γ_89-106_SlSN9 and γ_47-64_SlSN10, corresponded to the γ-core-containing regions of tomato snakins SlSN2, SlSN9, and SlSN10, and one peptide TkSN1_39-57_ to that of the wheat snakin TkSN1 (Table 14). All the snakin γ-core peptides were positively charged (net charge of +4 and +7) and hydrophilic. However, in contrast to defensin γ-core peptides, they did not adopt a β-hairpin conformation in the snakin 3D structure, which was predicted to lack β-structure and resemble a large hairpin consisting of two α-helices [46]. All the γ-core peptides of snakins possessed potent antimicrobial activity against plant and human pathogens. Among all tested tomato and wheat γ-core peptides, they showed the highest inhibitory activity and the broadest antimicrobial spectrum [46,53]. The wheat snakin γ-core peptide TkSN1_39-57_ was especially active against *C. neoformans* (IC_50_ = 6.0 μM) and *C. michiganensis* (IC_50_ = 12.0 μM). Of *Fusarium* species, TkSN1_39-57_ effectively inhibited *F. culmorum* (IC_50_ = 27.5 μM). The tomato snakin-derived peptides γ_48-65_SlSN2 and γ_89-106_SlSN9 were even more effective against *C. neoformans* (IC_50_ = 4.2 and 5.1 μM, respectively) than TkSN1_39-57_. *C. michiganensis* was also efficiently suppressed by these tomato peptides (Table 14). *Fusarium* species were more effectively inhibited by γ_48-65_SlSN2 (IC_50_ from 42.1 to 152.0 μM), although γ_89-106_SlSN9 and γ_47-64_SlSN10 were also highly active against particular *Fusarium* species. To study the mode of action of the snakin γ-core peptides γ_89-106_SlSN9 and TkSN1_39-57_, staining of *C. albicans* and *C. neoformans* cells with propidium iodide in the presence of these peptides was carried out, and analyzed by fluorescence microscopy [46,53]. It was shown that the peptides caused the permeabilization of the fungal membranes.

Analysis of the antimicrobial activity of the tomato and wheat nsLTP-derived peptides γ_56-72_SlLTPg2.4, γ_53-69_SlLTPg2.5, γ_70-86_SlLTPg2.8, TkLTPd5.6_59-75_, and TkLTP2.25_50-62_ showed that most of them were either inactive or displayed weak antimicrobial activity against the tested pathogens [46,53]. Only TkLTP2.25_50-62_ efficiently inhibited *C. neoformans* (IC_50_ = 45 μM). Low activity of the γ-cores of nsLTPs correlates with the negative charge or the absence of charge in the nsLTP-derived peptides (Table 15). The lack of antimicrobial activity in most nsLTP-derived peptides points to their signaling functions in the immune response.

The MEG (Maternally Expressed Gene)-derived peptide γ_92-104_SlMEG2 nearly completely inhibited the growth of *C. neoformans* and *C. michiganensis* at a high peptide concentration of 300 μM (Table 16) [46]. Since the role of MEG peptides in plant physiology is largely unknown, the discovery of antimicrobial activity in γ_92-104_SlMEG2 indicates their role in defense as antimicrobial agents.

The antimicrobial properties of the γ-core-containing peptides WAMP-G1 and WAMP-G2 of the wheat hevein-like peptide WAMP-2 were also explored [54]. The sequence of WAMP-G2 included the γ-core in D-isoform GFCGSGDAYC and four amino acid residues located N-terminally to the γ-core (Table 17). WAMP-G1 encompassed a putative γ-core in L2-isoform CGKYGFC, two N-terminal and three C-terminal amino acid residues. WAMP-G1 contained two antiparallel β-strands with an interposed turn region postulated to be a characteristic feature of the γ-core multidimensional signature [54]. In addition to the γ-core motif, the longer peptide WAMP-G2 contained all three aromatic residues of the chitin-binding site, while WAMP-G1 only had two residues (Tyr22 and Phe24).

The WAMP-2-derived γ-core peptides were assayed against seven fungal pathogens of plants, including *B. sorokiniana*, *A. alternata, Cladosporium cucumerinum, Parastagonospora nodorum,* and three *Fusarium* species (*F. oxysporum, F. culmorum, F. avenaceum*) (Table 17) [54]. WAMP-G2 inhibited the growth of all tested fungi, with *B. sorokiniana* and *A. alternata* being the most sensitive species. A comparison of the antimicrobial potential of WAMP-G1 and WAMP-G2 showed that the latter peptide was more active than WAMP-G1 against all tested pathogens. Taken that WAMP-G2 differed from WAMP-G1 by four amino acid residues from the C-terminus, including a conserved residue of the chitin-binding site Tyr31, this result shows that all three conserved aromatic residues of the chitin-binding site contribute to the antifungal activity of WAMP-G2.

In addition to antifungal activity, the ability of the γ-core peptides to increase the antifungal effect of the fungicide tebuconazole was assayed [54,55]. Treatments with the fungicide combined with WAMP-2 γ-core peptides inhibited spore germination of *B. sorokiniana*, *A. alternata, A. solani*, *F. culmorum*, *F. avenacenum*, *F. oxysporum,* and *P. nodorum* at a much greater level than the fungicide alone (except a combination of WAMP-G1 with tebuconazole against *A. alternata*), and the type of interactions between the fungicide and the peptides was either synergistic or additive, depending on the target fungus and concentration combinations. Compared to WAMP-G1, WAMP-G2 was shown to be a more active sensitizing fragment, synergistically enhancing the effect of sub-fungicidal tebuconazole dosages against all pathogens.

## 4. Discussion

Identification of molecular determinants of antimicrobial activity in AMPs is of vital importance for elucidation of their mode of action, which in turn is necessary for practical use in agriculture and medicine. In our review, we combined the available data regarding the activities of the γ-core peptides of different plant AMPs. Some γ-core sequences are widespread in defensins of a particular plant family. For example, the MsDef1 γ-core from the *M. sativa* defensin has close homologs in the Fabaceae family (VuDef1 and PvD1) (Figure S1). However, some γ-core sequences occur in AMPs of plants belonging to different families. The most spectacular example is the MtDef4 γ-core sequence found in defensins of Fabaceae, Solanaceae, Amaranthaceae, and Poaceae plants.

The results presented confirm the antimicrobial potential of the γ-core peptides derived from plant AMPs. This follows from the direct determination of their antimicrobial activity (Table 1, Table 2, Table 3, Table 4, Table 5, Table 6, Table 7, Table 8, Table 9, Table 10, Table 11, Table 12, Table 13, Table 14, Table 15, Table 16 and Table 17). Many γ-core peptides display activity not only against plant pathogens but against human pathogens as well, making them promising leads for pharmaceutical purposes. Antimicrobial assays showed that just as AMPs differ in their antimicrobial activity, so do their γ-cores.

Comparison of the antimicrobial activity of the γ-core peptides with that of the whole AMP molecules shows that γ-core peptides are, in the vast majority of cases, several-fold less active. Only single peptides, such as peptide 7 of OsAFP1, were claimed to exhibit the same efficiency as the parent peptide [49]. The reduced activity of the γ-core peptides compared to the parent peptides points to the existence of additional determinants of antimicrobial activity beyond the γ-core. In some cases, the α-core plays this role, as was shown for BhDef12 and BhDef23 α-core peptides of *B. hybrida* defensins and SmAP_α1-21_ and SmAP_α10-21_ of *S. marianum* defensin [25,38]. In some instances, the residues beyond the γ- and α-core regions were shown to be also important for activity, as exemplified by the radish defensins Rs-AFP1 and Rs-AFP2 [20].

### 4.1. Residues within γ-Core Sequences Responsible for Antimicrobial Activity

In a number of studies, the residues within the γ-core sequence responsible for activity have been identified. Thus, in the hexapeptide RGFRRR of the MtDef4 γ-core, the residues Phe37 and Arg38 were shown to be important for antimicrobial activity. Their substitution for Ala dramatically decreased activity [9]. The significance of an FR pair in the antifungal activity of the MtDef4 γ-core homologs was also shown for the wheat γ-core peptides TkDEFL1-11_55-68_ and TkDEFL1-32_55-68_ [53]. Phe is present in a number of other γ-core peptides (Figure S1); however, it remains unclear whether it is vital for their activity. Only in RsAFP2 Phe was shown to be important for the antifungal activity against *F. culmorum* [20]. However, most residues of the γ-core are not conserved in γ-cores of different AMPs. In the bi-domain MtDef5, His36 and Arg37 in MtDef5A and His93 and Arg94 in MtDef5B were demonstrated to be essential for antifungal activity [29]. In the RsAFP2 γ-core, beyond Phe40, such functional residues are Tyr38, Pro41 and Lys44 [20]; in the OsAFP1 γ-core motif peptide, Leu39 and Arg41 [50]; and in the maize γ-core peptide ES-d, Leu48, Cys49 and Tyr50 [51].

### 4.2. The Role of Net Charge in Antimicrobial Activity of γ-Core Peptides

Increasing the positive charge of the γ-core peptide usually enhances antimicrobial efficiency, as was clearly demonstrated for PvD1 variants γ_31-45_PvD1^++^ and γ_33-41_PvD1 (Table 5) [31]. A similar enhancement of the antimicrobial potency was observed in the α-core peptide BhDef12M (Table 2) [25].

Negatively charged or neutral peptides are mostly inactive or weakly active (e.g., nsLTP-derived peptides γ_53-69_SlLTPg2.5, γ_56-72_SlLTPg2.4, and γ_70-86_SlLTPg2.8, BhDef22, etc.) [25,46]. The importance of a positive charge for the antimicrobial activity of all γ-core peptides was argued by comparison of three OefDef1.1 variants harboring three or four neighboring Ala (Table 13) [10]. Their activity was similar or even higher than that of the wild-type peptide, although the charge of the γ-core motif in the Ala variants was lower than that of the parent peptide, suggesting that the activity was more dependent on hydrophobicity than on net charge [10].

### 4.3. The Role of Peptide’s Length and 3D Structure in Antimicrobial Activity

Regarding the length of the γ-core peptides, the results presented show that the longer peptides are usually more active than the shorter peptides, as follows, for example, from the comparison of antifungal activity of 9- and 15-mer γ-core peptides of PvD1 [31]. For Rs-AFP1, linear peptides with a length of 19 or 20 amino acids displayed the highest antifungal activity. Sequences of 16 amino acid residues or less showed lower levels of antifungal activity [22]. However, there exist certain exceptions: for example, the short positively charged peptide MBG07 of five amino acids derived from the C-terminal region of the Rs-AFP2 γ-core and the hexapeptide RGFRRR found in the γ-cores of different defensins [9,22]. Short peptides exhibiting antimicrobial activity are preferable for practical use due to low production costs; however, other peptide characteristics, such as cytotoxicity, stability, bioavailability, etc., should be additionally explored.

The 3D structure has not been solved for any γ-core peptide. We have carried out molecular modeling of the wheat and tomato γ-core peptides [46,53]. Most peptides have a helical region and are amphipathic, with hydrophobic and polar residues located on opposite sides of the helix. Some were predicted to exist in a random coil conformation. Analyzing the predicted 3D structures, we did not find any correlation between the spatial structure of peptides and their antimicrobial activity. The most potent defensin- and snakin-derived peptides display either α-helical or random-coil conformation. However, similar 3D structures were predicted for the least active LTP- and thionin-derived peptides. The characteristic feature that seems important for the antimicrobial activity of short peptides is the charge of the peptide, which is much higher in the peptides with pronounced antimicrobial properties than in the virtually inactive peptides.

### 4.4. The Role of Cysteine Residues in Activity

The role of cysteine residues in the activity of γ-core peptides remains ambiguous. The γ-core signature GXCX_3-9_C harbors two cysteine residues, which are highly reactive and prone to oxidation with the formation of inter and intramolecular disulfide bonds. In order to prevent oxidation, in a number of studies, cysteines in the γ-core motif peptides were substituted with alanine or α-aminobutyric acid. The comparison of the activities of cysteine-containing peptides with those carrying α-aminobutyric acid made for Rs-AFP2 peptides showed that the activity varied depending on the peptide, possibly reflecting differences in the mode of action [22]. Short α-aminobutyric acid-containing peptides showed lower potency than cysteine-containing peptides, while a longer peptide, MBG02, was even more active than the cysteine-containing MBG01. Reduced activity in the variants with cysteine substitutions might indicate that peptide multimerization is a necessary step in the mechanism of action. In the studies of wheat γ-core peptides, the activity of the loop peptides with and without the adjacent cysteine residues demonstrated that for most tested pathogens, the peptides with cysteine residues were more active than those without them [53]. However, for some pathogens, the opposite trend was observed.

### 4.5. The Mode of Action

The data regarding the mode of action of the γ-core motif peptides is fragmentary. Studies of the antimicrobial activity of the MtDef4 and MtDef5A γ-core peptides towards filamentous fungi showed that they suppressed spore germination, germ tube elongation, and mycelial growth in *P. medicaginis* and *F. solani* without causing morphological changes of spores or hyphae [23]. In *N. crassa*, the ubiquitous RGFRRR peptide derived from the γ-core of MtDef4 selectively inhibited cell fusion [26]. It was also necessary for MtDef4 internalization into the cells of *F. graminearum* [28]. It was shown that this peptide interacts with high affinity with phosphatidic acid, and this interaction mediates the transport of MtDef4 into the fungal cell walls. MtDef5A γ-core peptide did not act directly on the bacterial OM and possibly had an intracellular target [27].

Pathogen growth inhibition by γ-core peptides starts with membrane permeabilization [9,38,43,46,53]. Subsequent events were investigated only for a limited number of peptides. The fungicidal effect on *C. buinensis* cells of the γ-core peptide γ_33-41_PvD1^++^ occurred due to programmed cell death via an apoptotic pathway involving induction of oxidative stress and dysfunction of mitochondria and activation of metacaspases [31]. The γ-core peptide DD from VuDef1 acted similarly on the parasite *L. amazonensis* [32]. Studies of the mode of action of the α-core and γ-core peptides derived from the DefSm2-D defensin point to their distinct modes of action [38]. Studies of the mode of action of the γ-core peptide BcDef1 on the bacterial human pathogen *S. epidermidis* showed that it caused membrane and cell wall damage by altering membrane potential and permeability. The peptide also displayed antioxidant activity without causing cytotoxicity to normal mammalian cells. The γ-core peptide Atr-DEF2(G39-C54) affected the composition of OM in *E. coli* cells and triggered iron deficiency [40]. The mode of action of the γ-core peptides on different fungi is different, as was shown for the ES-d peptide from the defensin ZmES4 [52].

In addition to antimicrobial activity, a regulatory role was shown for the tomato SolyC07g007760 defensin γ-core peptide SolyC. The SolyC peptide down-regulated the level of the proinflammatory cytokines TNF-α and IFN-γ, thus acting as an antimicrobial and anti-inflammatory agent [44].

### 4.6. Synergism with Antimycotics

An interesting finding regarding γ-core peptides is that they act in synergy with AMPs and potentiate the action of antimycotics. Thus, the γ-core motif peptides 8 and 9 of Rs-AFP2, in combination with the parent peptide, enhanced the activity of Rs-AFP2 more than two-fold [21]. The γ-core peptide HsLin06 of HsAFP1 defensin acted synergistically with caspofungin in preventing *C. albicans* biofilm formation [48]. The γ-core peptides of the wheat WAMP-2 were shown to enhance the inhibitory effect of tebuconazole, used in agriculture to combat harmful cereal diseases. The type of interactions between the fungicide and the peptide fragment was either synergistic or additive [54,55].

## 5. Conclusions

The γ-core motif peptides of plant AMPs exhibit inhibitory activity against plant and human bacterial and fungal pathogens that makes them attractive scaffolds for the development of novel anti-infective agents for medicine and agriculture. At least some of them are multifunctional and combine antimicrobial and immunomodulatory functions, thus broadening the spectrum of practical applications. While γ-core motif peptides preserve all the advantages of AMPs: they are natural, broad-spectrum, and effective, their additional benefits include easy structure modification to enhance antimicrobial potency and other physicochemical characteristics and cost-effective production. Besides, an important advantage of γ-core derived peptides is that they act synergistically with antimycotics and fungicides, so combinations of peptides with conventionally used antifungal agents can be suggested as an effective strategy to reduce the doses of aggressive chemicals. However, to be used as next-generation antimicrobials, they should be additionally explored for cytotoxicity, chemical, and physical stability, bioavailability, etc.

We believe that further research into the γ-core motif peptides will satisfy the increased interest in peptides in pharmaceutical science and agriculture.

## Figures and Tables

**Table 1:** Rs-AFP2 and derived peptides: sequence, net charge at pH 7.0, and antimicrobial activity. Cysteine residues are shown in red, and substituted amino acids are in green. B—α-aminobutyric acid, c—D-cysteine. The α-core is underlined with a dotted line, and the γ-core is with a solid line. Note: nd—not determined, *—both figures are given in the original article [20].

Peptide	Amino Acid Sequence	Net Charge at pH 7.0
	Antimicrobial Activity	Reference
Rs-AFP2	**QKLC** **QRPSGTWSGV** **C** **GNNNA** **C** **KNQ** **C** **IRLEKARHGS** **C** **NYVFPAHK** **C** **I** **C** **YFP** **C**	+6
	Inhibited the growth of *Fusarium culmorum* with MIC = 3 µg/mL (IC_50_ = 2.9 (2.1) * µg/mL in a low ionic strength medium SMF− and IC_50_ = 8.1 (4.6)* µg/mL in the same medium with salts SMF+); *Alternaria brassicola* with MIC = 3 µg/mL (IC_50_ = 3.2 µg/mL in a low ionic strength medium SMF− and IC_50_ > 50 µg/mL in the same medium with salts SMF+); *Ascochyta pisi* with MIC = 6 µg/mL (IC_50_ = 1.9 µg/mL in a low ionic strength medium SMF− and IC50 > 50 µg/mL in the same medium with salts SMF+); *Botrytis cinerea* with MIC = 12 µg/mL (IC_50_ = 1.8 µg/mL in a low ionic strength medium SMF− and IC50 > 50 µg/mL in the same medium with salts SMF+); *Verticillium dahliae* with MIC = 3 µg/mL (IC_50_ = 1 µg/mL in a low ionic strength medium SMF− and IC_50_ = 11 µg/mL in the same medium with salts SMF+). No activity at 400 µg/mL against bacterial species *Aeromonas hydrophila* ssp. *proteoytica*, *Pseudomonas fluorescens*, *Escherichia coli*, *Bacillus cereus*, *B. subtilis*, *Staphylococcus aureus.* Inhibited the growth of *Nectria hematococca* with IC_50_ = 2 µg/mL in a low ionic strength medium SMF− and IC_50_ = 48 µg/mL in the same medium with salts SMF+ against; Inhibited the growth of *Phoma betae* with IC_50_ = 0.9 µg/mL in a low ionic strength medium SMF− and IC_50_ = 14 µg/mL in the same medium with salts SMF+ against; Inhibited the growth of *F. culmorum* with IC_50_ = 5 µg/mL/.	[20,21,22]
Peptide 1	**QKL** **C** **QRPSGTWSGV** **C**	+2
	Inhibited the growth of *F. culmorum* and *A. brassicola* with MIC = 250 µg/mL; *V. dahliae* with MIC = 250 µg/mL; *A. pisi* and *B. cinerea* with MIC > 500 µg/mL. No activity at 400 µg/mL against bacterial species *A. hydrophila* ssp. *proteoytica*, *P. fluorescens*, *E. coli*, *B. cereus*, *B. subtilis*, *S. aureus*.	[21]
Peptide 2	**QRPSGTWSGV** **C** **GNNN**	+1
	Inhibited the growth of *F. culmorum*, *A. brassicola*, *A. pisi*, *B. cinerea*, *V. dahliae* with MIC > 500 µg/mL. No activity at 400 µg/mL against bacterial species *A. hydrophila* ssp. *proteoytica*, *P. fluorescens*, *E. coli*, *B. cereus*, *B. subtilis*, *S. aureus*.	[21]
Peptide 3	**GTWSGV** **C** **GNNNA** **C** **KN**	+1
	Inhibited the growth of *F. culmorum* and *A. brassicola* with MIC = 250 µg/mL; *V. dahliae* with MIC = 250 µg/mL. No activity at 500 µg/mL against *A. pisi* and *B. cinerea*. No activity at 400 µg/mL against bacterial species *A. hydrophila* ssp. *proteoytica*, *P. fluorescens.*	[21]
Peptide 4	**GV** **C** **GNNNA** **C** **KNQ** **C** **IR**	+2
	Inhibited the growth of *F. culmorum* with MIC = 250−500 µg/mL; *A. brassicola* with MIC = 500 µg/mL; *B. cinerea* with MIC = 250 µg/mL; *V. dahliae* with MIC = 125 µg/mL. No activity at 500 µg/mL against *A. pisi*. No activity at 400 µg/mL against bacterial species *A. hydrophila* ssp. *proteoytica*, *P. fluorescens*, *E. coli*, *B. cereus, B. subtilis, S. aureus*.	[21]
Peptide 5	**NNNA** **C** **KNQ** **C** **IRLEKA**	+2
	Inhibited the growth of *F. culmorum* with MIC = 250 µg/mL; *A. brassicola* with MIC = 125 µg/mL; *A. pisi* with MIC = 500 µg/mL; *B. cinerea* with MIC = 500 µg/mL; *V. dahliae* with MIC = 250 µg/mL. No activity at 400 µg/mL against bacterial species *A. hydrophila* ssp. *proteoytica*, *E. coli, B. cereus*, *S. aureus*; Inhibited the growth of *P. fluorescens*, *B. subtilis* with MIC = 400 µg/mL.	[21]
Peptide 6	**C** **KNQ** **C** **IRLEKARHGS**	+3
	Inhibited the growth of *F. culmorum* with MIC = 45 µg/mL; *A. brassicola* with MIC = 60 µg/mL; *A. pisi* with MIC = 125 µg/mL; *B. cinerea* with MIC = 250 µg/mL; *V. dahliae* with MIC = 30 µg/mL. No activity at 400 µg/mL against bacterial species *A. hydrophila* ssp. *proteoytica*, *E. coli*, *B. cereus*, *S. aureus*; Inhibited the growth of *P. fluorescens*, *B. subtilis* with MIC = 100 µg/mL.	[21]
Peptide 7	**C** **IRLEKARHGS** **C** **NYV**	+2
	Inhibited the growth of *F. culmorum* with MIC = 60 µg/mL; *A. brassicola* with MIC = 60 µg/mL; *A. pisi* with MIC = 250 µg/mL; *B. cinerea* with MIC = 250 µg/mL; *V. dahlia* with MIC = 30 µg/mL. No activity at 400 µg/mL against bacterial species *A. hydrophila* ssp. *proteoytica, P. fluorescens, E. coli, B. cereus, B. subtilis, S. aureus*.	[21]
Peptide 8	**EKARHGS** **C** **NYVFPAH**	+1
	Inhibited the growth of *F. culmorum* with MIC = 45 µg/mL; *A. brassicola* with MIC = 30 µg/mL; *A. pisi* with MIC = 125 µg/mL; *B. cinerea* with MIC = 250 µg/mL; *V. dahliae* MIC = 30 µg/mL. No activity at 400 µg/mL against bacterial species *A. hydrophila* ssp. *proteoytica*, *P. fluorescens, E. coli, B. cereus, B. subtilis, S. aureus*.	[21]
Peptide 9	**HGS** **C** **NYVFPAHK** **C** **I** **C**	+1
	Inhibited the growth of *F. culmorum* with MIC = 30 µg/mL; *A. brassicola* with MIC = 60 µg/mL; *A. pisi* with MIC = 250 µg/mL; *B. cinerea* with MIC = 250 µg/mL; *V. dahliae* MIC = 30 µg/mL. No activity at 400 µg/mL against bacterial species *A. hydrophila* ssp. *proteoytic*a, *P. fluorescens, E. coli, B. cereus, B. subtilis, S. aureus.*	[21]
Peptide 10	**NYVFPAHK** **C** **I** **C** **YFP** **C**	+1
	Inhibited the growth of *F. culmorum* and *V. dahliae* with MIC = 500 µg/mL; *A. brassicola, A. pisi* and *B. cinerea* with MIC > 500 µg/mL. No activity at 400 µg/mL against bacterial species *A. hydrophila* ssp. *proteoytica*, *P. fluorescens*, *E. coli, B. cereus, B. subtilis, S. aureus*.	[21]
C36-C45	**C** **NYVFPAHK** **C**	+1
	Inhibited the growth of *F. culmorum* with MIC = 150 µg/mL.	[21]
C36-C45(Y38A)	**C** **N** **A** **VFPAHK** **C**	+1
	Inhibited the growth of *F. culmorum* with MIC > 400 µg/mL.	[21]
Rs-AFP2 (γ-core)	**GS** **C** **NYVFPAHK** **C** **I** **C** **YFP**	+1
	IC_50_ = 5.3 μM against *Fusarium tricinctum*. No activity at 30 μg/mL against *Phoma medicaginis* STC and WS-2 strains, *F. oxysporum* f. sp. *medicaginis* 7F-3 and 31F-3 strains, *Colletotrichum trifolli* FG-1 and WS-5 strains, *Aphanomyces euteiches*, *F. redolens*, *F. incarnatum, F.solani*; Inhibited the growth of *E. coli*, *Pseudomonas syringae* pv. *syringae*, *Sinorhizobium meliloti.*	[23]
Rs-AFP2(Q5M)	**QKL** **C** **M** **RPSGTWSGV** **C** **GNNNA** **C** **KNQ** **C** **IRLEKARHGS** **C** **NYVFPAHK** **C** **I** **C** **YFP** **C**	+6
	IC_50_ = 4.1 µg/mL in a low ionic strength medium SMF− and IC_50_ = 5.4 µg/mL in the same medium with salts SMF+ against *F. culmorum.*	[20]
Rs-AFP2(T10G)	**QKL** **C** **QRPSG** **G** **WSGV** **C** **GNNNA** **C** **KNQ** **C** **IRLEKARHGS** **C** **NYVFPAHK** **C** **I** **C** **YFP** **C**	+6
	IC_50_ = 11 µg/mL in a low ionic strength medium SMF− and IC_50_ > 100 µg/mL in the same medium with salts SMF+ against *F. culmorum.*	[20]
Rs-AFP2(G16M)	**QKL** **C** **QRPSGTWSGV** **C** **M** **NNNA** **C** **KNQ** **C** **IRLEKARHGS** **C** **NYVFPAHK** **C** **I** **C** **YFP** **C**	+6
	IC_50_ = 2.2 µg/mL in a low ionic strength medium SMF− and IC_50_ = 5.0 µg/mL in the same medium with salts SMF+ against *F. culmorum.*	[20]
Rs-AFP2(A31W)	**QKL** **C** **QRPSGTWSGV** **C** **GNNNA** **C** **KNQ** **C** **IRLEK** **W** **RHGS** **C** **NYVFPAHK** **C** **I** **C** **YFP** **C**	+6
	IC_50_ = 30 µg/mL in a low ionic strength medium SMF− and IC_50_ > 100 µg/mL in the same medium with salts SMF+ against *F. culmorum.*	[20]
Rs-AFP2(Y38G)	**QKL** **C** **QRPSGTWSGV** **C** **GNNNA** **C** **KNQ** **C** **IRLEKARHGS** **C** **N** **G** **VFPAHK** **C** **I** **C** **YFP** **C**	+6
	IC_50_ = 42 µg/mL in a low ionic strength medium SMF− and IC_50_ > 200 µg/mL in the same medium with salts SMF+ against *F. culmorum.*	[20]
Rs-AFP2(F40M)	**QKL** **C** **QRPSGTWSGV** **C** **GNNNA** **C** **KNQ** **C** **IRLEKARHGS** **C** **NYV** **M** **PAHK** **C** **I** **C** **YFP** **C**	+6
	IC_50_ = 16 µg/mL in a low ionic strength medium SMF− and IC_50_ = 54 µg/mL in the same medium with salts SMF+ against *F. culmorum.*	[20]
Rs-AFP2(P41∆)	**QKL** **C** **QRPSGTWSGV** **C** **GNNNA** **C** **KNQ** **C** **IRLEKARHGS** **C** **NYVF** **∆** **AHK** **C** **I** **C** **YFP** **C**	+6
	IC_50_ = 100 µg/mL in a low ionic strength medium SMF− and IC_50_ > 200 µg/mL in the same medium with salts SMF+ against *F. culmorum.*	[20]
Rs-AFP2(K44Q)	**QKL** **C** **QRPSGWSGV** **C** **GNNNA** **C** **KNQ** **C** **IRLEKARHGS** **C** **NYVFPAH** **Q** **C** **I** **C** **YFP** **C**	+5
	IC_50_ = 3.6 µg/mL in a low ionic strength medium SMF− and IC_50_ = 36 µg/mL in the same medium with salts SMF+ against *F. culmorum.*	[20]
Rs-AFP2(Y48I)	**QKL** **C** **QRPSGTWSGV** **C** **GNNNA** **C** **KNQ** **C** **IRLEKARHGS** **C** **NYVFPAHK** **C** **I** **C** **I** **FP** **C**	+6
	IC_50_ = 9.3 µg/mL in a low ionic strength medium SMF− and IC_50_ = 11 µg/mL in the same medium with salts SMF+ against *F. culmorum.*	[20]
Rs-AFP2(P7R)	**QKL** **C** **QR** **R** **SGTWSGV** **C** **GNNNA** **C** **KNQ** **C** **IRLEKARHGS** **C** **NYVFPAHK** **C** **I** **C** **YFP** **C**	+7
	IC_50_ = 6.8 µg/mL in a low ionic strength medium SMF− and IC_50_ = 8.8 µg/mL in the same medium with salts SMF+ against *F. culmorum.*	[20]
Rs-AFP2(G9R)	**QKL** **C** **QRPS** **R** **TWSGV** **C** **GNNNA** **C** **KNQ** **C** **IRLEKARHGS** **C** **NYVFPAHK** **C** **I** **C** **YFP** **C**	+7
	IC_50_ = 3 µg/mL in a low ionic strength medium SMF− and IC_50_ = 3.3 µg/mL in the same medium with salts SMF+ against *F. culmorum.*	[20]
Rs-AFP2(S12R)	**QKL** **C** **QRPSGTW** **R** **GV** **C** **GNNNA** **C** **KNQ** **C** **IRLEKARHGS** **C** **NYVFPAHK** **C** **I** **C** **YFP** **C**	+7
	IC_50_ = 3.5 µg/mL in a low ionic strength medium SMF− and IC_50_ = 20 µg/mL in the same medium with salts SMF+ against *F. culmorum.*	[20]
Rs-AFP2(I26R)	**QKL** **C** **QRPSGTWSGV** **C** **GNNNA** **C** **KNQ** **C** **R** **RLEKARHGS** **C** **NYVFPAHK** **C** **I** **C** **YFP** **C**	+7
	IC_50_ = 7.2 µg/mL in a low ionic strength medium SMF− and IC_50_ = 9.6 µg/mL in the same medium with salts SMF+ against *F. culmorum.*	[20]
Rs-AFP2(L28R)	**QKL** **C** **QRPSGTWSGV** **C** **GNNNA** **C** **KNQ** **C** **IR** **R** **EKARHGS** **C** **NYVFPAHK** **C** **I** **C** **YFP** **C**	+7
	IC_50_ = 6.4 µg/mL in a low ionic strength medium SMF− and IC_50_ >100 µg/mL in the same medium with salts SMF+ against *F. culmorum.*	[20]
Rs-AFP2(N37R)	**QKL** **C** **QRPSGTWSGV** **C** **GNNNA** **C** **KNQ** **C** **IRLEKARHGS** **C** **R** **YVFPAHK** **C** **I** **C** **YFP** **C**	+7
	IC_50_ = 2.8 µg/mL in a low ionic strength medium SMF− and IC_50_ = 7.0 µg/mL in the same medium with salts SMF+ against *F. culmorum.*	[20]
Rs-AFP2(V39R)	**QKL** **C** **QRPSGTWSGV** **C** **GNNNA** **C** **KNQ** **C** **IRLEKARHGS** **C** **NY** **R** **FPAHK** **C** **I** **C** **YFP** **C**	+7
	IC_50_ = 4.0 (2.2)* µg/mL in a low ionic strength medium SMF− and IC_50_ = 3.2 (2.3)* µg/mL in the same medium with salts SMF+ against *F. culmorum*; IC_50_ = 2.5 µg/mL in a low ionic strength medium SMF− and IC_50_ = 50 µg/mL in the same medium with salts SMF+ against *A. brassicicola*; IC_50_ = 2.0 µg/mL in a low ionic strength medium SMF− and IC_50_ > 50 µg/mL in the same medium with salts SMF+ against *A. pisi*; IC_50_ = 1.6 µg/mL in a low ionic strength medium SMF− and IC_50_ > 50 µg/mL in the same medium with salts SMF+ against *B. cinerea*; IC_50_ = 0.4 µg/mL in a low ionic strength medium SMF− and IC_50_ = 2.3 µg/mL in the same medium with salts SMF+ against *V. dahlia*; IC_50_ = 2.1 µg/mL in a low ionic strength medium SMF− and IC_50_ = 9 µg/mL in the same medium with salts SMF+ against *N. hematococca*; IC_50_ = 1.4 µg/mL in a low ionic strength medium SMF− and IC_50_ = 40 µg/mL in the same medium with salts SMF+ against *P. betae.*	[20]
Rs-AFP2(A42R)	**QKL** **C** **QRPSGTWSGV** **C** **GNNNA** **C** **KNQ** **C** **IRLEKARHGS** **C** **NYVFP** **R** **HK** **C** **I** **C** **YFP** **C**	+7
	IC_50_ = 4.2 µg/mL in a low ionic strength medium SMF− and IC_50_ = 18 µg/mL in the same medium with salts SMF+ against *F. culmorum.*	[20]
Rs-AFP2(I46R)	**QKL** **C** **QRPSGTWSGV** **C** **GNNNA** **C** **KNQ** **C** **IRLEKARHGS** **C** **NYVFPAHK** **C** **R** **C** **YFP** **C**	+7
	IC_50_ = 12 µg/mL in a low ionic strength medium SMF− and IC_50_ > 40 µg/mL in the same medium with salts SMF+ against *F. culmorum.*	[20]
Rs-AFP2(F49R)	**QKL** **C** **QRPSGTWSGV** **C** **GNNNA** **C** **KNQ** **C** **IRLEKARHGS** **C** **NYVFPAHK** **C** **I** **C** **Y** **R** **P** **C**	+7
	IC_50_ = 22 µg/mL in a low ionic strength medium SMF− and IC_50_ = 23 µg/mL in the same medium with salts SMF+ against *F. culmorum.*	[20]
MBG01	**ARHGS** **C** **NYVFPAHK** **C** **I** **C** **YF**	+2
	Inhibited the growth of *F. culmorum* with IC_50_ = 33 µg/mL.	[22]
MBG02	**ARHGS** **B** **NYVFPAHK** **B** **I** **B** **YF**	nd
	Inhibited the growth of *F. culmorum* with IC_50_ = 8 µg/mL.	[22]
MBG03	**C** **RHGS** **B** **NYVFPAHK** **B** **I** **B** **Y** **C**	nd
	Inhibited the growth of *F. culmorum* with IC_50_ = 17 µg/mL.	[22]
MBG04	**C** **RHGS** **c** **NYVFPAHK** **c** **I** **B** **Y** **C**	nd
	Inhibited the growth of *F. culmorum* with IC_50_ = 15 µg/mL.	[22]
MAT09	**HGS** **C** **NYVFPAHK** **C** **I** **C**	+1
	Inhibited the growth of *F. culmorum* with IC_50_ = 41 µg/mL.	[22]
MBG05	**HGS** **B** **NYVFPAHK** **B** **I** **B**	nd
	Inhibited the growth of *F. culmorum* with IC_50_ > 400 µg/mL.	[22]
MBG06	**ARHGS** **C**	+1
	Inhibited the growth of *F. culmorum* with IC_50_ = 159 µg/mL.	[22]
MBG08	**ARHGS** **B**	nd
	Inhibited the growth of *F. culmorum* with IC_50_ > 400 µg/mL.	[22]
MBG07	**HK** **C** **I** **C** **Y**	+1
	Inhibited the growth of *F. culmorum* with IC_50_ = 16µg/mL.	[22]
MBG09	**HK** **B** **I** **B** **Y**	nd
	Inhibited the growth of *F. culmorum* with IC_50_ > 400 µg/mL.	[22]
MAT02	**QRPSGTWSGV** **C** **GNNN**	+1
	Inhibited the growth of *F. culmorum* with IC_50_ > 400 µg/mL.	[22]
overlapping 13- to 15-mer peptides derived from Rs-AFP2(26−49)	**IRLEKARHGS** **B** **NYVFPAHK** **B** **I** **B** **YF**	nd
	15-mer peptide 26−40 had the lowest IC_50_ = 51 µg/mL against *F. culmorum*.	[22]
overlapping 16- to 20-mer peptides derived from Rs-AFP2(26–49)	**IRLEKARHGS** **B** **NYVFPAHK** **B** **I** **B** **YF**	nd
	Peptide 30–49: IC_50_ = 5 µg/mL against *F. culmorum*; Peptide 31–49: IC_50_ = 6 µg/mL against *F. culmorum*; Peptide 30–48: IC_50_ = 10 µg/mL against *F. culmorum*; Peptide 32–49: IC_50_ = 9 µg/mL against *F. culmorum.*	[22]

**Table 2:** BhDef1, BhDef2, and derived peptides: sequence, net charge at pH 7.0, and antimicrobial activity. Cysteine residues are shown in red, and substituted amino acids are shown in green. Gaps (−) were introduced to improve the alignment. The α-core is underlined with a dotted line, and the γ-core is with a solid line.

Peptide	Amino Acid Sequence	Net Charge at pH 7.0
	Antimicrobial Activity	Reference
BhDef1	**--------** **KL** ** C ** **ERGRG--TWSGV** ** C ** ** GNNNA ** ** C ** **KNQ** **C** **IRLEGAQHGS** ** C ** ** NNVFPAHK ** ** C ** **I** ** C ** **YFP** ** C **	+4
	**−**	[25]
BhDef11	**KL** **C** **ERGRG--TWSGV** **C** **G**	+2
	BhDef11 showed inhibitory activity against *Colletotrichum gloeosporioides* DoA c1060 at a concentration of 1.024 mg/mL with inhibition zone around 4 mm, and no activity against *C. gloeosporioides* DoA d0762 and *C. capsici* DoA c1511 at 1.024 mg/mL; it inhibited the growth of methicillin resistant *Staphylococcus aureus* (MRSA, ATCC 43300) with MIC_99_ = 2.23 mg/mL and the growth of *Salmonella* Typhi (ATCC 13311) with MIC_99_ = 2.02 mg/mL.	[25]
BhDef12	**GV** **C** **GNNNA** **C**	0
	No activity against *C. gloeosporioides* DoA c1060 at a concentration of 0.512 mg/mL and against *C. gloeosporioides* DoA d0762 and *C. capsici* DoA c1511 at 1.024 mg/mL.	[25]
BhDef12M	**GV** **C** **G** **RRR** **A** **C**	+3
	BhDef12M showed inhibitory activity against *C. gloeosporioides* DoA c1060 at a concentration of 0.52 mM (0.512mg/mL) with inhibition zone around 4 mm; no activity against *C. gloeosporioides* DoA d0762 and *C. capsici* DoA c1511 at 1.024 mg/mL; inhibited the growth of *S. aureus* (MRSA) with MIC_99_ = 0.93 mg/mL; inhibited the growth of *Salmonell*a Typhi with MIC_99_ = 2.00 mg/mL	[25]
BhDef13	**RLEGAQHGS** **C** **NNVFPAHK** **C**	+1
	BhDef13 showed inhibitory activity against *C. gloeosporioides* DoA c1060 at a concentration of 0.50 mM (1.024 mg/mL) with inhibition zone around 7 mm; no activity against *C. gloeosporioides* DoA d0762 and *C. capsici* DoA c1511 at 1.024 mg/mL, inhibited the growth of *S. aureus* (MRSA) with MIC_99_ = 2.26 mg/mL; inhibited the growth of *Salmonella* Typhi with MIC_99_ = 1.71 mg/mL.	[25]
BhDef14	**KL** **C** **ERGRG--TWSGV** **C** **GNNNA** **C** **KNQ** **C** **IR**	+4
	BhDef14 showed inhibitory activity against *C. gloeosporioides* DoA c1060 at a concentration of 0.36 mM (1.024 mg/mL) with inhibition zone around 3 mm. No activity against *C. gloeosporioides* DoA d0762 and *C. capsici* DoA c1511 at 1.024 mg/mL, inhibited the growth of *S. aureus* (MRSA) with MIC_99_ = 2.40 mg/mL; inhibited the growth of *Salmonella* Typhi with MIC_99_ = 1.88 mg/mL.	[25]
BhDef2	**EASALRGGKR** ** C ** **EKRNSSTSFSGV** ** C ** ** QYDNA ** ** C ** **MNQ** ** C ** **INLEGAQDGK** ** C ** ** NNAVPTPK ** ** C ** **I** ** C ** **YFP** ** C **	+2
	**−**	[25]
BhDef21	**KR** **C** **EKRNSSTSFSGV** **C** **Q**	+3
	No activity against *C. gloeosporioides* DoA c1060 at a concentration of 0.512 mg/mL; no activity against *C. gloeosporioides* DoA d0762 and *C. capsici* DoA c1511 at 1.024 mg/mL; inhibited the growth of *S. aureus* (MRSA) with MIC_99_ = 2.14 mg/mL; inhibited the growth of *Salmonella* Typhi with MIC_99_ = 1.93 mg/mL.	[25]
BhDef22	**NLEGAQDGK** **C** **NNAVPTPK** **C**	0
	No activity against *C. gloeosporioides* DoA c1060 at a concentration of 0.512 mg/mL; no activity against *C. gloeosporioides* DoA d0762 and *C. capsici* DoA c1511 at 1.024 mg/mL.	[25]
BhDef23	**GV** **C** **QYDNA** **C**	−1
	BhDef23 showed inhibitory activity against *C. gloeosporioides* DoA c1060 at a concentration of 1.05 mM (1.024 mg/mL) with inhibition zone around 10 mm; no activity against *C. gloeosporioides* DoA d0762 and *C. capsici* DoA c1511 at 1.024 mg/mL; inhibited the growth of *S. aureus* (MRSA) with MIC_99_ = 2.93 mg/mL; inhibited the growth of *Salmonella* Typhi with MIC_99_ = 1.77 mg/mL.	[25]

**Table 3:** MsDef1, MtDef4, and derived peptides: sequence, net charge at pH 7.0, and antimicrobial activity. Cysteine residues are shown in red, and substituted amino acids are shown in green. Gaps (−) were introduced to improve the alignment. The α-core is underlined with a dotted line, and the γ-core is with a solid line.

Peptide	Amino Acid Sequence	Net Charge at pH 7.0
	Antimicrobial Activity	Reference
MsDef1	**RT** **C** **ENLADKYRGP** **C** **FS-G-** **C** **DTH** **C** **TTKENAVSGR** **C** **R-DDFR** **C** **W** **C** **TKR** **C**	+3
	IC_50_ = 2−4 μM against *F. graminearum*; MsDef1 caused 41 ± 5% growth inhibition of *F. verticillioides* at 24 μM; 90% growth inhibition of *Aspergillus flavus* at concentrations of 12 to 48 µM; IC_50_ (germination) > 24.50 µM, IC_50_(CAT (conidial anastomosis tube) fusion) = 7.62 µM and IC_50_ (cell death) = 1.62 µM against *Neurospora crassa.*	[9,26]
MsDef1-R38Q	**RT** **C** **ENLADKYRGP** **C** **FS-G-** **C** **DTH** **C** **TTKENAVSGR** **C** **R-DDF** **Q** **C** **W** **C** **TKR** **C**	+2
	IC_50_ > 6 μM against *F. graminearum.*	[9]
MsDef1-γ4	**RT** **C** **ENLADKYRGP** **C** **FS-G-** **C** **DTH** **C** **TTKENAVSGR** **C** **R** **GFRR** **R** **C** **W** **C** **TKR** **C**	+7
	IC_50_ = 1.3−1.5 μM against *F. graminearum.*	[9]
GMA1-C	**GR** **C** **R-DDFR** **C** **W** **C** **TKR** **C**	+3
	IC_50_ = 14 μM against *F. graminearum*; GMA1-C inhibited more than 90% of *F. verticillioides* growth at 24 µM; 90% growth inhibition of *A. flavus* at concentrations of 24 µM; IC_50_ = 12.7 μM against *P. medicaginis* STC and IC_50_ = 14.8 μM against *P. medicaginis* WS-2; No activity at 30 μg/mL against *F. oxysporum* f. sp. *medicaginis* 7F-3 and 31F-3 strains, *C. trifolli* FG-1 and WS-5 strains, *A. euteiches*, *F. solani*, *F. tricinctum*, *F. redolens*, *F. incarnatum*; IC_50_ = 7.9 μM against *Xanthomonas alfalfae* subsp. *alfalfae*; IC_50_ = 8.8 μM against *P. syringae* pv. *syringae*; IC_50_(germination) = 8.54 µM, IC_50_(CAT fusion) = 6.46 µM and IC_50_ (cell death) = 24.15 µM against *N. crassa.*	[9,23,26]
GMA1	**GR** **C** **R-DDFR** **C**	+1
	IC_50_ > 192 μM against *F. graminearum*; IC_50_ (germination) > 80 µM, IC_50_ (CAT fusion) >80 µM and IC_50_ (cell death) > 80 µM against *N. crassa.*	[9,26]
GMA1-L	**R-DDFR**	0
	IC_50_ >96 μM against *F. graminearum*; IC_50_ (germination) > 80 µM, IC_50_ (CAT fusion) >80 µM and IC_50_ (cell death) > 80 µM against *N. crassa.*	[9,26]
ALP1	**GP** **C** **FS-G-** **C**	0
	IC_50_ >48 μM against *F. graminearum.*	[9]
MtDef4	**RT** **C** **ESQSHKFKGP** **C** **ASDHN** **C** **ASV** **C** **QT-ERFSGGR** **C** **RGFRRR** **C** **F** **C** **TTH** **C**	+6
	IC_50_ = 0.75−1 μM against *F. graminearum*; MtDef4 inhibited more than 90% of *F. verticillioides* growth at 12 µM; *A. flavus* growth inhibition of approximately 75% at 48 µM; IC_50_ = 0.3 μM against *P. medicaginis* STC and IC_50_ = 2.6 μM against *P. medicaginis* WS-2; IC_50_ = 0.7 μM against against *F. oxysporum* f. sp. *medicaginis* 7F-3 and IC_50_ = 1.9 μM against 31F-3 strain; no activity at 30 μg/mL against *A. euteiches, C. trifolli* FG-1 and WS-5 strains; IC_50_ = 0.6 μM against *X. alfalfae* subsp. alfalfae; IC_50_ = 0.4 μM against *P. syringae* pv. *syringae*; IC_50_ = 0.1 μM against *Clavibacter insidiosus*; IC_50_ (germination) = 0.65 µM, IC_50_ (CAT fusion) = 0.52 µM and IC_50_ (cell death) = 0.83 µM against *N. crassa.*	[9,23,26]
GMA4-C	**GR** **C** **RGFRRR** **C** **F** **C** **TTH** **C**	+5
	IC_50_ = 3 μM against *F. graminearum*; GMA4-C inhibited more than 90% of *F. verticillioides* growth at 3 µM; 90% growth inhibition of *A. flavus* at concentrations of 6 µM; IC_50_ = 7.3 μM against *P. medicaginis* STC and IC_50_ = 5.3 μM against *P. medicaginis* WS-2; IC_50_ = 7.1 μM against *F. oxysporum* f. sp. *medicaginis* 7F-3 and IC_50_ = 6.9 μM against 31F-3 strain. No activity at 30 μg/mL against *C. trifolli* FG-1 and WS-5 strains, *A. euteiches*, *F. redolens, F. incarnatum*; IC_50_ = 6.0 μM against *F. solani*; IC_50_ = 14.7 μM against *F. tricinctum*; IC_50_ = 11.4 μM against *X. alfalfae* subsp. *alfalfae*; IC_50_ = 3.4 μM against *P. syringae* pv. *syringae*; IC_50_ = 8.4 μM against *Serratia marcescens*; IC_50_ = 2.3 μM against *Enterobacter aerogenes*; IC_50_ = 2.7 μM against *Pseudomonas aeruginosa*; no activity at 30 μg/mL against *Enterococcus casseliflavus*; IC_50_ = 1.7-4.2 μM against *P. aeruginosa* strains; IC_50_ (germination) = 1.6 µM, IC_50_ (CAT fusion) = 1.37 µM and IC_50_ (cell death) = 2.21 µM against *N. crassa.*	[9,23,26,27]
GMA4	**GR** **C** **RGFRRR** **C**	+5
	IC_50_ = 3 μM against *F. graminearum*; IC_50_ (germination) = 2.20 µM, IC_50_ (CAT fusion) = 1.68 µM and IC_50_ (cell death) = 2.64 µM against *N. crassa.*	[9,26]
GMA4-L	**RGFRRR**	+4
	IC_50_ = 4 μM against *F. graminearum*; IC_50_ (germination) = 55.38 µM, IC_50_(CAT fusion) = 4.10 µM and IC_50_ (cell death) > 80 µM against *N. crassa.*	[9,26]
GMA4-L1	**RG** **A** **RRR**	+4
	IC_50_ > 96 μM against *F. graminearum.*	[9]
GMA4-L2	**RGF** **A** **RR**	+3
	IC_50_ > 96 μM against *F. graminearum.*	[9]
ALP4	**GP** **C** **ASDHN** **C**	−1
	IC_50_ > 48 μM against *F. graminearum.*	[9]
MtDef4^RGFRRR/AAAARR^	**RT** **C** **ESQSHKFKGP** **C** **ASDHN** **C** **ASV** **C** **QT-ERFSGGR** **C** **AAAA** **RR** **C** **F** **C** **TTH** **C**	+4
	Growth inhibition of *F. graminearum* by less than 10% at 3 µM.	[28]
MtDef4^RGFRRR/RGAARR^	**RT** **C** **ESQSHKFKGP** **C** **ASDHN** **C** **ASV** **C** **QT-ERFSGGR** **C** **RG** **AA** **RR** **C** **F** **C** **TTH** **C**	+5
	More than 90% growth inhibition of *F. graminearum* at 3 μM.	[28]
MtDef4^RGFRRR/RGFRAA^	**RT** **C** **ESQSHKFKGP** **C** **ASDHN** **C** **ASV** **C** **QT-ERFSGGR** **C** **RGFR** **AA** **C** **F** **C** **TTH** **C**	+4
	About 30% growth inhibition of *F. graminearum* at 3 μM.	[28]

**Table 4:** MtDef5 and derived peptides: sequence, net charge at pH 7.0, and antimicrobial activity. Cysteine residues are shown in red and substituted amino acids are in green. The MtDef5 defensin domains, MtDef5A and MtDef5B are connected by a 7-amino acid linker shown in italic. The α-core is underlined with a dotted line, and the γ-core is with a solid line.

Peptide	Amino Acid Sequence	Net Charge at pH 7.0
	Antimicrobial Activity	Reference
MtDef5	**KL** **C** **QKRSTTWSGP** **C** **LNTGN** **C** **KRQ** **C** **INVEHATFGA** **C** **HRQGFGFA** **C** **F** **C** **YKK** **C** ***APKKVEP*** **KL** **C** **ERRSKTWSGP** **C** **LISGN** **C** **KRQ** **C** **INVEHATSGA** **C** **HRQGIGFA** **C** **F** **C** **KKK** **C**	+16
	MtDef5 inhibited the growth of *F. graminearum* and *N. crassa* with an IC_50_ value of 0.25–0.3 µM (MIC of 0.70–0.75 µM). It also inhibited the growth of several other filamentous plant fungal pathogens including *F. verticilloides*, *F. thapsinum*, *Alternaria brassicicola*, *Colletotrichum higginsianum,* and *Botrytis cinerea*, *C. higginsianum* was the least sensitive pathogen among this group of fungal pathogens with an IC_50_ value of 1.75 µM (MIC of 3.0 µM); MtDef5 inhibited the growth of *Xanthomonas campestris* with MIC of 12 µM and *Clavibacter michiganensis* with MIC > 12 µM; IC_50_ = 1.5 μM against *P. medicaginis* STC and IC_50_ = 1.6 μM against *P. medicaginis* WS-2; IC_50_ = 0.8 μM against agaisnt *F. oxysporum* f. sp. *medicaginis* 7F-3 and IC_50_ = 1.3 μM against 31F-3 strain; no activity at 30 μg/mL against *C. trifolli* FG-1 and WS-5 strains, *A. euteiches*; IC_50_ = 0.1 μM against *P. syringae* pv. *syringae*; no activity at 30 μg/mL against *X. alfalfae* subsp. *alfalfae* and *Clavibacter insidiosus*.	[23,29,30]
MtDef5A	**KL** **C** **QKRSTTWSGP** **C** **LNTGN** **C** **KRQ** **C** **INVEHATFGA** **C** **HRQGFGFA** **C** **F** **C** **YKK** **C**	+7
	MtDef5A inhibited the growth of *F. graminearum* with an IC_50_ value of 0.75–1.0 µM (MIC of 1.5-3.0 µM); inhibited the growth of *X. campestris* with MIC of 12 µM and *C. michiganensis* with MIC > 12 µM.	[29,30]
MtDef5B	**KL** **C** **ERRSKTWSGP** **C** **LISGN** **C** **KRQ** **C** **INVEHATSGA** **C** **HRQGIGFA** **C** **F** **C** **KKK** **C**	+8
	MtDef5B inhibited the growth of *F. graminearum* with an IC_50_ value of 0.5–0.75 µM (MIC of 1.0–1.5 µM); inhibited the growth of *X. campestris* with MIC of 6 µM and *C. michiganensis* with MIC > 12 µM.	[29,30]
MtDef5^H36A, R37A/H93A, R94A^ (MtDef5_V1)	**KL** **C** **QKRSTTWSGP** **C** **LNTGN** **C** **KRQ** **C** **INVEHATFGA** **C** **AA** **QGFGFA** **C** **F** **C** **YKK** **C** ***APKKVEP*** **KL** **C** **ERRSKTWSGP** **C** **LISGN** **C** **KRQ** **C** **INVEHATSGA** **C** **AA** **QGIGFA** **C** **F** **C** **KKK** **C**	+14
	MtDef5_V1 almost completely lost its antifungal activity against *F. graminearum*; inhibited the growth of *X. campestris* and *C. michiganensis* with MIC > 12 µM.	[29,30]
MtDef5^Q38A, G39A/Q95A, G96A^ (MtDef5_V2)	**KL** **C** **QKRSTTWSGP** **C** **LNTGN** **C** **KRQ** **C** **INVEHATFGA** **C** **HR** **AA** **FGFA** **C** **F** **C** **YKK** **C** ***APKKVEP*** **KL** **C** **ERRSKTWSGP** **C** **LISGN** **C** **KRQ** **C** **INVEHATSGA** **C** **HR** **AA** **IGFA** **C** **F** **C** **KKK** **C**	+16
	MtDef5_V2 showed 2-fold reduction in its antifungal activity against *F. graminearum* relative to that of MtDef5.	[29]
MtDef5^F40A, G41A/I97A, G98A^ (MtDef5_V3)	**KL** **C** **QKRSTTWSGP** **C** **LNTGN** **C** **KRQ** **C** **INVEHATFGA** **C** **HRQG** **AA** **FA** **C** **F** **C** **YKK** **C** ***APKKVEP*** **KL** **C** **ERRSKTWSGP** **C** **LISGN** **C** **KRQ** **C** **INVEHATSGA** **C** **HRQG** **AA** **FA** **C** **F** **C** **KKK** **C**	+16
	MtDef5_V3 retained MtDef5 antifungal activity against *F. graminearum.*	[29]
MtDef5^F42A/F99A^ (MtDef5_V4)	**KL** **C** **QKRSTTWSGP** **C** **LNTGN** **C** **KRQ** **C** **INVEHATFGA** **C** **HRQGFG** **A** **A** **C** **F** **C** **YKK** **C** ***APKKVEP*** **KL** **C** **ERRSKTWSGP** **C** **LISGN** **C** **KRQ** **C** **INVEHATSGA** **C** **HRQGIG** **A** **A** **C** **F** **C** **KKK** **C**	+16
	MtDef5_V4 retained MtDef5 antifungal activity against *F. graminearum.*	[29]
MtDef5A (γ-core)	**GA** **C** **HRQGFGFA** **C** **F** **C** **YKK** **C**	+3
	IC_50_ = 19.5 μM against *P. medicaginis* STC and IC_50_ = 8.5 μM against *P. medicaginis* WS-2; IC_50_ = 4.1 μM against F. solani; no activity at 30 μg/mL agaisnt *F. oxysporum* f. sp. *medicaginis* 7F-3 and 31F-3 strains, *C. trifolli* FG-1 and WS-5 strains, *A. euteiches, F. tricinctum, F. redolens, F. incarnatum*; IC_50_ = 4.5 μM against *P. syringae* pv. *syringae*; no activity at 30 μg/mL against *X. alfalfae* subsp. *alfalfae*; IC_50_ = 6.0 μM against *S. marcescens*; IC_50_ = 2.8 μM against *E. aerogenes*; IC_50_ = 11.8 μM against *P. aeruginosa*; no activity at 30 μg/mL against *Enterococcus casseliflavus*; IC_50_ = 8.5–14.6 μM against *P. aeruginosa* strains.	[23,27]

**Table 5:** PvD1 and derived peptides: sequence, net charge at pH 7.0, and antimicrobial activity. Cysteine residues are shown in red and substituted amino acids are in green. The α-core is underlined with a dotted line, and the γ-core is with a solid line.

Peptide	Amino Acid Sequence	Net Charge at pH 7.0
	Antimicrobial Activity	Reference
PvD1	**KT** **C** **ENLADTYKGP** **C** **FTTGS** **C** **DDH** **C** **KNKEHLRSGR** **C** **RDDFR** **C** **W** **C** **TKN** **C**	+2
	Inhibited the growth of the yeasts, *C. albicans*, *C. parapsilosis*, *C. tropicalis*, *C. guilliermondii*, *Kluyveromyces marxiannus* and *Saccharomyces cerevisiae* and phytopathogenic fungi including *F. oxysporum*, *F. solani*, *F. lateritium* and *Rizoctonia solani*.	[31]
γ_31-45_PvD1	**RSGR** **A** **RDDFR** **A** **W** **A** **TK**	+3
	40% growth inhibition of *C. albicans* at 293.6 μM; 100% growth inhibition of *C. buinensis* at 293.6 μM.	[31]
γ_31-45_PvD1^++^	**RSGR** **A** **R** **RR** **FR** **A** **W** **A** **TK**	+7
	100% growth inhibition of *C. albicans* at 73.4 μM; 100% growth inhibition of *C. buinensis* at 18.35 μM.	[31]
γ_33-41_PvD1	**GR** **A** **RDDFR** **A**	+1
	No activity at 293.6 μM against *C. albicans*; 17% growth inhibition of *C. buinensis* at 293.6 μM.	[31]
γ_33-41_PvD1^++^	**GR** **A** **R** **RR** **FR** **A**	+5
	63% growth inhibition of *C. albicans* at 293.6 μM; 100% growth inhibition of *C. buinensis* at 36.7 μM.	[31]

**Table 6:** VuDef1 and derived peptides: sequence, net charge at pH 7.0, and antimicrobial activity. Cysteine residues are shown in red and substituted amino acids are in green. The α-core is underlined with a dotted line, and the γ-core is with a solid line.

Peptide	Amino Acid Sequence	Net Charge at pH 7.0
	Antimicrobial Activity	Reference
VuDef1	**KT** **C** **ENLADTYRGP** **C** **FTTGS** **C** **DDH** **C** **KNKEHLLSGR** **C** **RDDVR** **C** **W** **C** **TRN** **C**	+1
	51% growth inhibition of *Leishmania amazonensis* at 18.5 μM.	[32]
A_36,42,44_γ_32-46_VuDef (DD)	**LSGR** **A** **RDDVR** **A** **W** **A** **TR**	+2
	41% growth inhibition of *L. amazonensis* at 18.5 μM; no activity at 18.5 μM against *C. albicans, C. buinensis, C. tropicalis*, *Saccharomices cerevisiae, C. parapsilosis* and *C. pelliculosa.*	[32]
A_36,42,44_R_37,38_γ_32-46_VuDef (RR)	**LSGR** **A** **R** **RR** **VR** **A** **W** **A** **TR**	+6
	47.5, 100 and 72.1% growth inhibition of *C. albicans, C. buinensis*, and *C. tropicalis*, respectively, at 18.5 μM; for *C. tropicalis*, MIC_100_ and LD_100_ = 27.5 μM; no activity at 18.5 μM against *S. cerevisiae, C. parapsilosis* and *C. pelliculosa.*	[32]
D-A_36,42,44_R_37,38_γ_32-46_VuDef (D-RR)	**LSGR** **A** **R** **RR** **VR** **A** **W** **A** **TR**	+6
	84.9, 99.7, and 100% growth inhibition of *C. albicans, C. buinensis*, and *C. tropicalis*, respectively, at 18.5 μM; for *C. albicans*, MIC_100_ = 23 μM and LD_100_ = 36.5 μM; for *C. tropicalis*, MIC_100_ = 14 μM and LD_100_ = 23 μM; no activity at 18.5 μM against *S. cerevisiae, C. parapsilosis* and *C. pelliculosa.*	[32]
A_42,44_R_37,38_W_36,39_γ_32-46_VuDef (WR)	**LSGR** **W** **R** **RRW** **R** **A** **W** **A** **TR**	+6
	26.1, 96.2, 98.5, and 58.2% growth inhibition of *S. cerevisiae, C. albicans, C. buinensis*, and *C. tropicalis*, respectively, at 18.5 μM; for *C. albicans*, MIC_100_ = 18.5 μM and LD_100_ = 27.5 μM; no activity at 18.5 μM against *C. parapsilosis* and *C. pelliculosa.*	[32]

**Table 7:** DefSm2-D, Atr-DEF2, So-D2, BcDef, and derived peptides: sequence, net charge at pH 7.0, and antimicrobial activity. Cysteine residues are shown in red and substituted amino acids are in green. Gaps (−) were introduced to improve the alignment. The α-core is underlined with a dotted line, and the γ-core is with a solid line.

Peptide	Amino Acid Sequence	Net Charge at pH 7.0
	Antimicrobial Activity	Reference
DefSm2-D	**-----** **KL** ** C ** **EKPSKTWFGN** ** C ** ** GNPRH ** ** C ** **GDQ** ** C ** **KSWEGAVHGA** ** C ** ** HVRNGKHM ** ** C ** **F** ** C ** **YFN** ** C ** **PQAE**	+3
	**−**	[38]
SmAP_γ27-44_	**WEGAVHGA** **C** **HVRNGKHM** **C**	+1
	Inhibited the growth of *F. graminearum* with MIC = 20 µM.	[38]
SmAP_γ29-35_	**GAVHGA** **C**	0
	No activity at 100 μM against *F. graminearum*.	[38]
SmAP_α1-21_	**KL** **C** **EKPSKTWFGN** **C** **GNPRH** **C** **G**	+3
	Inhibited the growth of *F. graminearum* with MIC = 32 µM.	[38]
SmAP_α10-21_	**WFGN** **C** **GNPRH** **C** **G**	+1
	Inhibited the growth of *F. graminearum* with MIC = 70 µM.	[38]
Atr-DEF2	**-----** **RI** ** C ** **ESASYRFKGI** ** C ** ** VSRTN ** ** C ** **ANV** ** C ** **KT-EGFPGGR** ** C ** ** RG--FRRR ** ** C ** **F** ** C ** **YKH** ** C ** **A**	+9
	**−**	[40]
Atr-DEF2(G39-C54)	**GR** **C** **RG--FRRR** **C** **F** **C** **YKH** **C**	+6
	IC_50_ = 9 μM against *E. coli*; IC*50* = 68 μM against *Klebsiella pneumoniae*.	[40]
So-D2	**GIFSSRK** ** C ** **KTPSKTFKGI** ** C ** ** TRDSN ** ** C ** **DTS** ** C ** **R-YEGYPAGD** ** C ** ** KG--IRRR ** ** C ** **M** ** C ** **SKP** ** C **	+8
	EC_50_ (effective concentration for 50% inhibition) = 1 μM against *C. michiganensis*; EC_50_ = 2 μM against *Ralstonia solanacearum*; EC_50_ = 0.2 μM against *F. culmorum*; EC_50_ = 11 μM against *F. solani*; MIC = 7.5 μg/mL against *P. aeruginosa* ATCC 27853; MIC = 7.5 μg/mL against *C. albicans* ATCC 64124. It also inhibited *E. coli* ATCC 25922 (MIC = 30 μg/mL) and *K. pneumoniae* ATCC 700603 (MIC = 30 μg/mL).	[41,42]
So-D2 (γ-core)	**GD** **C** **KG--IRRR** **C** **M** **C** **SKP** **L**	+4
	IC_50_ = 6.4 μM against *P. medicaginis* STC and IC_5_0 = 6.1 μM against *P. medicaginis* WS-2; IC_50_ = 33.1 μM against *F. oxysporum* f. sp. *medicaginis* 7F-3; IC_50_ = 13.8 μM against *F. solani*; No activity at 30 μg/mL against *F. oxysporum* f. sp. *medicaginis* 31F-3, *F. redolens, F. incarnatum, F. tricinctum, C. trifolli* FG-1 and WS-5 strains, *A. euteiches*; IC_50_ = 25.9 μM against *P. syringae* pv. *syringae*; IC_50_ = 19.3 μM against *X. alfalfae* subsp. *alfalfae*; No activity at 30 μg/mL against *C. insidiosus*	[23]
BcDef	**-----** **RH** ** C ** **ESQSQRFKGT** ** C ** ** LSEKN ** ** C ** **ASV** ** C ** **E-TEGFSGGD** ** C ** ** RG--LRRR ** ** C ** **F** ** C ** **TRP** ** C **	+4
	**−**	[43]
BcDef1	**FSGGD** **C** **RG--LRRR** **C** **F** **C** **TR**	+4
	Inhibited the growth of Gram-negative *E. coli* ATCC25922 with MIC > 251.21 µM; *E. coli* O157 with MIC = 229.09 µM; *P. aeruginosa* with MIC > 251.21 µM; *Vibrio cholerae* with MIC = 125.61 µM; *Shigella sonnei* with MIC = 125.61 µM; *Staphylococcus typhimurium* with MIC = 31.40 µM; Gram-positive *Enterococcus faecalis* with MIC = 251.21 µM; *B. cereus* with MIC = 251.21 µM; *Staphylococcus aureus* ATCC25923 and MRSA with MIC >251.21 µM; *Staphylococcus epidermidis* with MIC = 15.70 µM.	[43]

**Table 8:** Solyc07g007760, SlDEFL2, SlDEFL4, and derived peptides: sequence, net charge at pH 7.0, and antimicrobial activity. Cysteine residues are shown in red and substituted amino acids are in green. The α-core is underlined with a dotted line, and the γ-core is with a solid line.

Peptide	Amino Acid Sequence	Net Charge at pH 7.0
	Antimicrobial Activity	Reference
Solyc07g007760	**RH** **C** **ESLSHRFKGP** **C** **VSDKN** **C** **ASV** **C** **ETERFSGGN** **C** **RGFRRR** **C** **F** **C** **TKP** **C**	+6
	**−**	[44]
SolyC	**FSGGN** **C** **RGFRRR** **C** **F** **C** **TK**	+5
	Inhibited the growth of Gram-negative *E. coli* ATCC25922 and *Salmonella enterica* serovar Parathyphi with MIC = 15 µg/mL; *Helicobacter pylori* with MIC = 10−15 µg/mL; inhibited the growth of Gram-positive *Staphylococcus aureus* A170, *S. epidermidis* and *Listeria monocytogenes* with MIC = 40 µg/mL; 15% and 13% growth inhibition of *Lactobacillum plantarum* and *L. paracasei* at 50 μg/mL, respectively.	[44]
SolyC-t	**GN** **C** **RGFRRR** **C** **F** **C** **TK**	+5
	Inhibited the growth of Gram-negative *S. enterica* serovar Parathyphi B and *H. pylori* with MIC = 15 µg/mL; inhibited the growth of Gram-positive *S. aureus* with MIC = 50 µg/mL; *S. epidermidis* and *L. monocytogenes* with MIC = 80 µg/mL.	[45]
SolyC1	**FSGGN** **C** **RGFRRR** **C** **F** **S** **TK**	+5
	Inhibited the growth of Gram-negative *S. enterica* serovar Parathyphi B with MIC = 40 µg/mL; *H. pylori* with MIC = 15 µg/mL; Inhibited the growth of Gram-positive *S. aureus* with MIC = 50 µg/mL; *S. epidermidis* with MIC = 100 µg/mL; *L. monocytogenes* with MIC = 80 µg/mL.	[45]
SolyC1-t	**GN** **C** **RGFRRR** **C** **F** **S** **TK**	+5
	Inhibited the growth of Gram-negative *S. enterica* serovar Parathyphi B with MIC = 80 µg/mL; *H. pylori* with MIC = 15 µg/mL; inhibited the growth of Gram-positive *S. aureus* with MIC = 50 µg/mL; *S. epidermidis* with MIC = 100 µg/mL; *L. monocytogenes* with MIC = 80 µg/mL.	[45]
SolyC2	**FSGGN** **C** **RGFRRR** **S** **F** **C** **TK**	+5
	Inhibited the growth of Gram-negative *S. enterica* serovar Parathyphi B and *H. pylori* with MIC = 15 µg/mL; inhibited the growth of Gram-positive *S. aureus* with MIC = 80 µg/mL; *S. epidermidis* with MIC = 100 µg/mL; *L. monocytogenes* with MIC = 80 µg/mL.	[45]
SolyC2-t	**GN** **C** **RGFRRR** **S** **F** **C** **TK**	+5
	Inhibited the growth of Gram-negative *S. enterica* serovar Parathyphi B with MIC = 40 µg/mL; *H. pylori* with MIC = 20 µg/mL; inhibited the growth of Gram-positive *S. aureus* with MIC = 80 µg/mL; *S. epidermidis* with MIC = 100 µg/mL; *L. monocytogenes* with MIC = 80 µg/mL	[45]
SolyC1-ox (C6–C13)	**FSGGN** **C** **RGFRRR** **C** **F** **S** **TK**	+5
	Inhibited the growth of Gram-negative *S. enterica* serovar Parathyphi B and *H. pylori* with MIC = 15 µg/mL; inhibited the growth of Gram-positive *S. aureus* with MIC = 80 µg/mL; *S. epidermidis* with MIC = 100 µg/mL; *L. monocytogenes* with MIC = 80 µg/mL.	[45]
SolyC1-t-ox (C6–C13)	**GN** **C** **RGFRRR** **C** **F** **S** **TK**	+5
	Inhibited the growth of Gram-negative *S. enterica* serovar Parathyphi B and *H. pylori* with MIC = 15 µg/mL; inhibited the growth of Gram-positive *S. aureus* with MIC = 50 µg/mL; *S. epidermidis* with MIC = 100 µg/mL; *L. monocytogenes* with MIC = 80 µg/mL.	[45]
SolyC2-ox (C6–C15)	**FSGGN** **C** **RGFRRR** **S** **F** **C** **TK**	+5
	Inhibited the growth of Gram-negative *S. enterica* serovar Parathyphi B with MIC = 10 µg/mL; *H. pylori* with MIC = 20 µg/mL; inhibited the growth of Gram-positive *S. aureus* and *S. epidermidis* with MIC = 100 µg/mL; *L. monocytogenes* with MIC = 80 µg/mL.	[45]
SolyC2-t-ox (C6–C15)	**GN** **C** **RGFRRR** **S** **F** **C** **TK**	+5
	Inhibited the growth of Gram-negative *S. enterica* serovar Parathyphi B and *H. pylori* with MIC = 20 µg/mL; inhibited the growth of Gram-positive *S. aureus* with MIC = 80 µg/mL; *S. epidermidis* with MIC = 100 µg/mL; *L. monocytogenes* with MIC = 80 µg/mL.	[45]
SlDEFL2	**RT** **C** **ESQSHRFKGP** **C** **VSEKN** **C** **ASV** **C** **ETEGFSGGD** **C** **RGFRRR** **C** **F** **C** **TRP** **C**	+4
	**−**	[46]
γ_58-74_SlDEFL2	**FSGGD** **C** **RGFRRR** **C** **F** **C** **TR**	+4
	IC_50_ = 11.5 μM against *Cryptococcus neoformans*; IC_50_ = 19.8 μM against *Clavibacter michiganensis*; IC_50_ = 44.8 μM against *F. culmorum*; IC_50_ = 165.8 μM against *F. oxysporum*; 97% growth inhibition of *C. albicans* at 300 μM; 85% growth inhibition of *Pseudomonas savastanoi* at 300 μM; 48% growth inhibition of *Pectobacterium carotovorum* at 300 μM; 31% growth inhibition of *Botrytis cinerea* at 300 μM; no activity against *F. solani, F. verticillioides, Bipolaris sorokiniana* at 300 μM.	[46]
SlDEFL4	**R** **T** **C** **ESQSHHFKGN** **C** **LSDTN** **C** **GSV** **C** **RTEGFTGGN** **C** **RGFRRR** **C** **F** **C** **TRN** **C**	+5
	**−**	[46]
γ_58-74_SlDEFL4	**FTGGN** **C** **RGFRRR** **C** **F** **C** **TR**	+5
	IC_50_ = 8.1 μM against *C. neoformans*; IC_50_ = 21.5 μM against *C. michiganensis*; IC_50_ = 42.3 μM against *F. culmorum*; IC_50_ = 124.8 μM against *F. oxysporum*; 97% growth inhibition of *C. albicans* at 300 μM; 100% growth inhibition of *P. savastanoi* at 300 μM; 81% growth inhibition of *P. carotovorum* at 300 μM; 45% growth inhibition of *B. cinerea* at 300 μM; No activity against *F. solani*, *F. verticillioides, B. sorokiniana* at 300 μM.	[46]

**Table 9:** HsAFP1 and derived peptides: sequence, net charge at pH 7.0, and antimicrobial activity. Cysteine residues are shown in red and substituted amino acids are in green. B—α-aminobutyric acid. The α-core is underlined with a dotted line, and the γ-core is with a solid line. Note: nd—not determined.

Peptide	Amino Acid Sequence	Net Charge at pH 7.0
	Antimicrobial Activity	Reference
HsAFP1	**DGVKL** **C** **DVPSGTWSGH** **C** **GSSSK** **C** **SQQ** **C** **KDREHFAYGGA** **C** **HYQFPSVK** **C** **F** **C** **KRQ** **C**	+3
	IC_50_ = 0.45 μM against *F. culmorum*; MIC_50_ = 18.00 μM against planktonic *C. albicans* cultures; BIC_50_ (biofilm formation) = 11.00 μM against *C. albicans*.	[48]
HsLin01	**DGVKL** **B** **DVPSGTWSGH** **B** **GSSSK** **B** **S**	nd
	BIC_50_ > 175 μM against *C. albicans*.	[48]
HsLin02	**DVPSGTWSGH** **B** **GSSSK** **B** **SQQ** **B** **KDR**	nd
	BIC_50_ > 175 μM against *C. albicans*.	[48]
HsLin03	**WSGH** **B** **GSSSK** **B** **SQQ** **B** **KDREHFAYG**	nd
	BIC_50_ = 96.78 μM against *C. albicans*.	[48]
HsLin04	**SSSK** **B** **SQQ** **B** **KDREHFAYGGA** **B** **HYQ**	nd
	BIC_50_ > 175 μM against *C. albicans*.	[48]
HsLin05	**QQ** **B** **KDREHFAYGGA** **B** **HYQFPSVK** **B**	nd
	BIC_50_ = 160.00 μM against *C. albicans*.	[48]

**Table 10:** OsAFP1 and derived peptides: sequence, net charge at pH 7.0, and antimicrobial activity. Cysteine residues are shown in red and substituted amino acids are in green. The α-core is underlined with a dotted line, and the γ-core is with a solid line.

Peptide	Amino Acid Sequence	Net Charge at pH 7.0
	Antimicrobial Activity	Reference
OsAFP1	**RH** **C** **LSQSHRFKGM** **C** **VSSNN** **C** **ANV** **C** **RTESFPDGE** **C** **KSHGLERK** **C** **F** **C** **KKV** **C**	+5
	IC_50_ = 2 μM against *C. albicans* (MIC = 4 μM); MIC = 4 μM and 16 μM against *Saccharomyces cerevisiae* By4742 and S288C strains, respectively; MIC > 32 μM against Gram-negative bacteria *Porphyromonas gingivalis* and *E. coli*; MIC > 32 μM against Gram-positive bacteria *Streptococcus mutans*, *Staphylococcus aureus, Propionibacterium acnes*; IC_50_ = 0.99 μg/mL against *Pyricularia oryzae*; IC_50_ = 1.48 μg/mL against *Rhizoctonia solani*; IC_50_ = 3.75 μg/mL against *Gibberella fujikuroi*; IC_50_ > 30 μg/mL against bacteria *Burkholderia plantari*, *B. glumae* and *Acidovorax evenae*.	[49,50]
OsAFP1 (K35A)	**RH** **C** **LSQSHRFKGM** **C** **VSSNN** **C** **ANV** **C** **RTESFPDGE** **C** **A** **SHGLERK** **C** **F** **C** **KKV** **C**	+4
	IC_50_ = 15 μM against *C. albicans*.	[50]
OsAFP1 (H37A)	**RH** **C** **LSQSHRFKGM** **C** **VSSNN** **C** **ANV** **C** **RTESFPDGE** **C** **KS** **A** **GLERK** **C** **F** **C** **KKV** **C**	+5
	IC_50_ = 17 μM against *C. albicans*.	[50]
OsAFP1 (L39A)	**RH** **C** **LSQSHRFKGM** **C** **VSSNN** **C** **ANV** **C** **RTESFPDGE** **C** **KSHG** **A** **ERK** **C** **F** **C** **KKV** **C**	+5
	IC_50_ > 32 μM against *C. albicans*.	[50]
OsAFP1 (R41A)	**RH** **C** **LSQSHRFKGM** **C** **VSSNN** **C** **ANV** **C** **RTESFPDGE** **C** **KSHGLE** **A** **K** **C** **F** **C** **KKV** **C**	+4
	IC_50_ > 32 μM against *C. albicans*.	[50]
OsAFP1 (K42A)	**RH** **C** **LSQSHRFKGM** **C** **VSSNN** **C** **ANV** **C** **RTESFPDGE** **C** **KSHGLER** **A** **C** **F** **C** **KKV** **C**	+4
	IC_50_ = 20 μM against *C. albicans*.	[50]
Peptide 1	**RH** **C** **LSQSHRF**	+2
	IC_50_ = 6 μM against *C. albicans*; IC_50_ = 0.41 μg/mL against *P. oryzae*.	[49,50]
Peptide 2	**SHRFKGM** **C** **VS**	+2
	IC_50_ = 19 μM against *C. albicans*; IC_50_ = 0.87 μg/mL against *P. oryzae*.	[49,50]
Peptide 3	**VSSNN** **C** **ANV**	0
	IC_50_ > 25 μM against *C. albicans*; IC_50_ > 30 μg/mL against *P. oryzae*.	[49,50]
Peptide 4	**SNN** **C** **ANV** **C** **RTE**	0
	IC_50_ > 25 μM against *C. albicans*; IC_50_ > 30 μg/mL against *P. oryzae*.	[49,50]
Peptide 5	**RTESFPDGE**	−2
	IC_50_ > 25 μM against *C. albicans*; IC_50_ > 30 μg/mL against *P. oryzae*.	[49,50]
Peptide 6	**FPDGE** **C** **KSHG**	−1
	IC_50_ > 25 μM against *C. albicans*; IC_50_ > 30 μg/mL against *P. oryzae*.	[49,50]
Peptide 7	**KSHGLERK** **C** **F**	+2
	IC_50_ = 10 μM against *C. albicans*; IC_50_ = 0.84 μg/mL against *P. oryzae*.	[49,50]
Peptide 8	**ERK** **C** **F** **C** **KKV** **C**	+3
	IC_50_ = 13 μM against *C. albicans*; IC_50_ = 1.42 μg/mL against *P. oryzae*.	[49,50]

**Table 11:** ZmES1, ZmES4, and derived peptides: sequence, net charge at pH 7.0, and antimicrobial activity. Cysteine residues are shown in red and substituted amino acids are in green. The γ-core is underlined with a solid line.

Peptide	Amino Acid Sequence	Net Charge at pH 7.0
	Antimicrobial Activity	Reference
ZmES1	**RD** **C** **LTQSTRLPGHL** **C** **VRSDY** **C** **AIG** **C** **RAEGKGYTGGR** **C** **LISPIPLDGIL** **C** **Y** **C** **VKP** **C** **PSNTTT**	+3
	66.7% germination inhibition of *F. graminearum* at 90 μM; 55.9% germination inhibition of *Ustilago maydis* at 90 μM.	[52]
ZmES4	**RD** **C** **LTQSTRLPGHL** **C** **VRSDY** **C** **AIG** **C** **RAEGKGYTGGR** **C** **LISPITLDGIL** **C** **Y** **C** **VKP** **C** **TSTTTK**	+4
	67.8% germination inhibition of *F. graminearum* at 90 μM; 56.4% germination inhibition of *U. maydis* at 90 μM.	[52]
mES4	**RD** **C** **LTQSTRLPGHL** **C** **VRSDY** **C** **AIG** **C** **RAEGKGYTGGR** **C** **Q** **ISPITL** **AV** **I** **Q** **C** **Y** **C** **VKP** **C** **TSTTTK**	+5
	Significant inhibition was not observed.	[52]
ES-a	**RD** **C** **LTQSTRLPGHL** **C** **V**	+1
	Significant inhibition was not observed.	[52]
ES-b	**L** **C** **VRSDY** **C** **AIG** **C** **R**	+1
	Significant inhibition was not observed.	[52]
ES-c	**G** **C** **RAEGKGYTGGR** **C** **L**	+2
	77.1% germination inhibition of *F. graminearum* at 90 μM; 80.9% germination inhibition of *U. maydis* at 90 μM.	[52]
ES-d	**R** **C** **LISPITLDGIL** **C** **Y**	0
	79.3% germination inhibition of *F. graminearum* at 90 μM; 78.7% germination inhibition of *U. maydis* at 90 μM.	[52]
mES-d1	**R** **C** **Q** **ISPITL** **AV** **I** **Q** **C** **Y**	+1
	Significant inhibition was not observed.	[52]
mES-d2	**R** **C** **L** **AAAA** **TLDGIL** **C** **Y**	0
	Significant inhibition was not observed.	[52]
ES-e	**L** **C** **Y** **C** **VKP** **C** **TSTTTK**	+2
	Significant inhibition was not observed.	[52]

**Table 12:** TkDEFL1s and derived peptides: sequence, net charge at pH 7.0, and antimicrobial activity. Cysteine residues are shown in red. Gaps (−) were introduced to improve the alignment. The α-core is underlined with a dotted line, and the γ-core is with a solid line.

Peptide	Amino Acid Sequence	Net Charge at pH 7.0
	Antimicrobial Activity	Reference
TkDEFL1-11	**RI** **C** **TGKSQHHSFP** **C** **ISDKS** **C** **TKT** **C** **LGEHGAKWTAGY** **C** **K--ISR-** **C** **T** **C** **QRE** **C**	+5
	**−**	[53]
TkDEFL1-11_55-68_	**GY** **C** **K---ISR-** **C** **T** **C** **QRE** **C**	+2
	11% germination inhibition *C. neoformans* at 300 μM; 17% germination inhibition of *C. albicans* at 300 μM; 11% germination inhibition of *P. carotovorum* at 300 μM; No activity at 300 μM against *F. culmorum, F. oxysporum, F. solani, F. verticillioides, C. michiganensis, P. savastanoi*.	[53]
TkDEFL1-32	**RI** **C** **TGKSQHHSFP** **C** **ISDKS** **C** **TKT** **C** **LGEHGAKWTAGY** **C** **K---FRR-** **C** **T** **C** **QRE** **C**	+6
	**−**	[53]
TkDEFL1-32_55-68_	**GY** **C** **K---FRR-** **C** **T** **C** **QRE** **C**	+3
	IC_50_ = 42.9 μM against *C. neoformans* (100% germination inhibition at 80 μM); 92% germination inhibition of *C. albicans* at 300 μM; IC_50_ = 38.5 μM against *C. michiganensis* (76% germination inhibition at 300 μM); IC_50_ = 97.8 μM against *F. culmorum* (79% germination inhibition at 150 μM); IC_50_ = 89.3 μM against *F. oxysporum* (71% germination inhibition at 300 μM); 39% germination inhibition of *P. savastanoi* at 300 μM; 56% germination inhibition of *P. carotovorum* at 300 μM; no activity at 300 μM against *F. solani, F. verticillioides*.	[53]
TkDEFL1-12	**KI** **C** **RQRSAGFKGP** **C** **LSDKN** **C** **AQV** **C** **LQER---WGGGN** **C** **DG--PFRR** **C** **K** **C** **IRQ** **C**	+7
	**−**	[53]
TkDEFL1-12_62-77_	**GN** **C** **DG--PFRR** **C** **K** **C** **IRQ** **C**	+3
	IC_50_ = 37.3 μM against *C. neoformans* (97% germination inhibition at 100 μM); 5% germination inhibition of *C. albicans* at 300 μM; IC_50_ = 69.1 μM against *C. michiganensis* (89% germination inhibition at 300 μM); 71% germination inhibition of *F. culmorum* at 300 μM; 62% germination inhibition of *F. oxysporum* at 300 μM; 45% germination inhibition of *F. solani* at 300 μM; 6% germination inhibition of *P. savastanoi* at 300 μM; 30% germination inhibition of *P. carotovorum* at 300 μM; no activity against *F. verticillioides*.	[53]
TkDEFL1-16	**RT** **C** **ESRSHRFRGP** **C** **VRRSN** **C** **ANV** **C** **KTEG-FP--DGK** **C** **RG--FRRR** **C** **F** **C** **TTH** **C** **HH**	+9
	**−**	[53]
TkDEFL1-16_65-82_	**GK** **C** **RG--FRRR** **C** **F** **C** **TTH** **C** **HH**	+5
	IC_50_ = 4.4 μM against *C. neoformans* (100% germination inhibition at 20 μM); IC_50_ = 14.6 μM against *C. albicans* (96% germination inhibition at 20 μM); IC_50_ = 14.6 μM against *C. michiganensis* (91% germination inhibition at 20 μM); IC_50_ = 20.7 μM against *F. culmorum* (82% germination inhibition at 30 μM); IC_50_ = 12.1 μM against *F. oxysporum* (81% germination inhibition at 30 μM); IC_50_ = 52.5 μM against *F. solani* (74% germination inhibition at 150 μM); IC_50_ = 48.4 μM against *F. verticillioides* (59% germination inhibition at 150 μM); IC_50_ = 56.2 μM against *P. savastanoi* (100% germination inhibition at 100 μM); IC_50_ = 70.7 μM against *P. carotovorum* (100% germination inhibition at 100 μM).	[53]
TkDEFL1-20	**RT** **C** **LSQSHKFKGT** **C** **LSNSN** **C** **AGV** **C** **RTEN-FP--DGE** **C** **NTHLVERK** **C** **Y** **C** **KRT** **C**	+4
	**−**	[53]
TkDEFL1-20_65-82_	**GE** **C** **NTHLVERK** **C** **Y** **C** **KRT** **C**	+2
	74% germination inhibition of *C. neoformans* at 300 μM; 14% germination inhibition of *C. albicans* at 300 μM; 94% germination inhibition of *C. michiganensis* at 300 μM; 48% germination inhibition of *F. culmorum* at 300 μM; 53% germination inhibition of *F. oxysporum* at 300 μM; 31% germination inhibition of *P. carotovorum* at 300 μM; no activity at 300 μM against *F. solani*, *F. verticillioides, P. savastanoi*.	[53]
TkDEFL1-23	**RT** **C** **LSQSHKFKGT** **C** **LSNSN** **C** **AGV** **C** **RTEN-FP--DGE** **C** **NSHRLERK** **C** **Y** **C** **KRT** **C**	+5
	**−**	[53]
TkDEFL1-23_65-82_	**GE** **C** **NSHRLERK** **C** **Y** **C** **KRT** **C**	+3
	IC_50_ = 39.8 μM against *C. neoformans* (98% germination inhibition at 100 μM); IC_50_ = 57.1 μM against *C. michiganensis* (93% germination inhibition at 300 μM); IC_50_ = 46 μM against *F. culmorum* (75% germination inhibition at 75 μM); IC_50_ = 56.7 μM against *F. oxysporum* (70% germination inhibition at 150 μM); 28% germination inhibition of *P. savastanoi* at 300 μM; 29% germination inhibition of *P. carotovorum* at 100 μM; No activity at 300 μM against *C. albicans, F. solani, F. verticillioides*.	[53]
TkDEFL1-40	**RT** **C** **LSQSHKFKGT** **C** **LSNSN** **C** **AGV** **C** **RTEN-FP--DGE** **C** **NSHRLERK** **C** **F** **C** **KRT** **C**	+5
	**−**	[53]
TkDEFL1-40_65-82_	**GE** **C** **NSHRLERK** **C** **F** **C** **KRT** **C**	+3
	IC_50_ = 39.0 μM against *C. neoformans* (98% germination inhibition at 100 μM); IC_50_ = 51.6 μM against *C. michiganensis* (88% germination inhibition at 300 μM); IC_50_ = 59.3 μM against *F. culmorum* (78% germination inhibition at 150 μM); IC_50_ = 49.9 μM against *F. oxysporum* (78% germination inhibition at 150 μM); 32% germination inhibition of *P. savastanoi* at 300 μM; 19% germination inhibition of *P. carotovorum* at 300 μM; No activity at 300 μM against *C. albicans, F. solani, F. verticillioides*.	[53]
TkDEFL1-36	**RD** **C** **LSQSHKFKGA** **C** **ISSSN** **C** **AGV** **C** **RTEN-FP--DGE** **C** **HTHNFARK** **C** **F** **C** **KRA** **C**	+4
	**−**	[53]
TkDEFL1-36_65-82_	**GE** **C** **HTHNFARK** **C** **F** **C** **KRA** **C**	+3
	IC_50_ = 16.8 μM against *C. neoformans* (100% germination inhibition at 50 μM); 97% germination inhibition of *C. albicans* at 300 μM; IC_50_ = 48.6 μM against *C. michiganensis* (100% germination inhibition at 100 μM); IC_50_ = 38.3 μM against *F. culmorum* (80% germination inhibition at 50 μM); IC_50_ = 52.4 μM against *F. oxysporum* (80% germination inhibition at 70 μM);45% germination inhibition of *F. solani* at 300 μM; 55% germination inhibition of *F. verticillioides* at 180 μM; 54% germination inhibition of *P. carotovorum* at 300 μM; No activity against *P. savastanoi* at 300 μM.	[53]
TkDEFL4-4	**EQSISYKSLDQAHQA** **C** **PKHGT** **C** **APRGFSYTRGAK** **C** **IFYNGE** **C** **LG**	+2
	**−**	[53]
TkDEFL4-4_46-57_	**APRGFSYTRGAK**	+3
	40% germination inhibition of *C. neoformans* at 300 μM; 18% germination inhibition of *C. albicans* at 300 μM; 19% germination inhibition of *F. culmorum* at 300 μM; 8% germination inhibition of *F. oxysporum* at 300 μM; 24% germination inhibition of *F. solani* at 300 μM; 28% germination inhibition of *F. verticillioides* at 300 μM; 21% germination inhibition of *P. savastanoi* at 300 μM; 12% germination inhibition of *P. carotovorum* at 300 μM; no activity at 300 μM against *C. michiganensis*.	[53]
TkDEFL4-8	**-----------** **GTTHAIPVPTLRGIEDDDVGFAEREE--AAYP-RRRV** **LYGDQYISYKGVQASRPA** ** C ** **S--GS** ** C ** ** AGRGQPYT-GSG ** ** C ** **QAIFG-** ** C ** **HGR**	+1
	**−**	[53]
TkDEFL4-8_82-94_	**C** **AGRGQPYT-GSG** **C**	+1
	8% germination inhibition of *C. neoformans* at 300 μM; 17% germination inhibition of *C. albicans* at 300 μM; 14% germination inhibition of *C. michiganensis* at 300 μM; 10% germination inhibition of *F. oxysporum* at 300 μM; 15% germination inhibition of *F. solani* at 300 μM; 26% germination inhibition of *F. verticillioides* at 300 μM; 19% germination inhibition of *P. savastanoi* at 300 μM; 42% germination inhibition of *P. carotovorum* at 300 μM; no activity at 300 μM against *F. culmorum*.	[53]
TkDEFL4-8_83-93_	**AGRGQPYT-GSG**	+1
	3% germination inhibition of *C. neoformans* at 300 μM; 26% germination inhibition of *C. albicans* at 300 μM; 11% germination inhibition of *C. michiganensis* at 300 μM; 17% germination inhibition of *F. oxysporum* at 300 μM; 35% germination inhibition of *F. solani* at 300 μM; 13% germination inhibition of *F. verticillioides* at 300 μM; 5% germination inhibition of *P. savastanoi* at 300 μM; 12% germination inhibition of *P. carotovorum* at 300 μM; no activity at 300 μM against *F. culmorum*.	[53]
TkDEFL4-37	**AALARIDAAAAVMPTSSATWMKLEDGVAPELLGSTA---VDLEGHRRV** **LAS-TSITASSLNPNKAA** ** C ** **TRT--** ** C ** ** PARGRPYT-GRA ** ** C ** **LRRYQ-** ** C ** **RQGQ**	+6
	**−**	[53]
TkDEFL4-37_90-102_	**C** **PARGRPYT-GRA** **C**	+3
	100% germination inhibition of *C. neoformans* at 300 μM; 38% germination inhibition of *C. albicans* at 300 μM; 24% germination inhibition of *F. culmorum* at 300 μM; 31% germination inhibition of *F. oxysporum* at 300 μM; 34% germination inhibition of *F. verticillioides* at 150 μM; 38% germination inhibition of *P. savastanoi* at 300 μM; 36% germination inhibition of *P. carotovorum* at 300 μM; no activity at 300 μM against *C. michiganensis* and *F. solani*.	[53]
TkDEFL4-37_91-101_	**ARGRPYT-GRA**	+3
	10% germination inhibition of *C. neoformans* at 300 μM; 29% germination inhibition of *C. albicans* at 300 μM; 29% germination inhibition of *F. culmorum* at 300 μM; 18% germination inhibition of *F. oxysporum* at 300 μM; 25% germination inhibition of *F. verticillioides* at 300 μM; 14% germination inhibition of *P. savastanoi* at 300 μM; no activity at 300 μM against *C. michiganensis*, *F. solani* and *P. carotovorum*.	[53]
TkDEFL4-20	**DISAGFAASGAAYSIDAAVRQLMSPSSMKLEDGVDPEFSVDLEVHRRV** **LAG---ISPGALSRNRPA** ** C ** **P--GA** ** C ** ** PAPGGSYT-NRG ** ** C ** **QKKYQ-** ** C ** **RG**	+2
	**−**	[53]
TkDEFL4-20_86-110_	**A** **C** **P--GA** **C** **PAPGGSYT-NRG** **C** **QKKYQ-** **C** **R**	+4
	95% germination inhibition of *C. neoformans* at 300 μM; 11% germination inhibition of *C. albicans* at 300 μM; 91% germination inhibition of *C. michiganensis* at 300 μM; 60% germination inhibition of *F. culmorum* at 150 μM; 54% germination inhibition of *F. oxysporum* at 150 μM; 14% germination inhibition of *P. carotovorum* at 300 μM; no activity at 300 μM against *F. solani*, *F. verticillioides* and *P. savastanoi*.	[53]
TkDEFL4-20_92-102_	**PAPGGSYT-NRG**	+1
	15% germination inhibition of *C. neoformans* at 300 μM; 16% germination inhibition of *C. albicans* at 300 μM; 9% germination inhibition of *C. michiganensis* at 300 μM; 6% germination inhibition of *F. oxysporum* at 300 μM; 14% germination inhibition of *F. solani* at 300 μM; 5% germination inhibition of *F. verticillioides* at 300 μM; no activity at 300 μM against *F. culmorum*, *P. savastanoi* and *P. carotovorum*.	[53]
HvDEFL4-1	**----------------------------** **AAFAGGTASIDMAAAVHRRI** **LAD-PGLGSGVYNANNAA** ** C ** **GSQ--** ** C ** ** AGHGKRYT-GRG ** ** C ** **DSFYG-** ** C ** **RSKPP**	+4
	**−**	[53]
HvDEFL4-1_67-77_	** AGHGKRYT-GRG**	+3
	100% germination inhibition of *C. neoformans* at 300 μM; 25% germination inhibition of *C. albicans* at 300 μM; 34% germination inhibition of *F. culmorum* at 300 μM; 32% germination inhibition of *F. verticillioides* at 300 μM; No activity at 300 μM against *C. michiganensis*, *F. oxysporum, F. solani, P. savastanoi* and *P. carotovorum*.	[53]

**Table 13:** OefDef1.1 and derived peptides: sequence, net charge at pH 7.0, and antimicrobial activity. Cysteine residues are shown in red, and substituted amino acids are in green. Gaps (−) were introduced to improve the alignment. The α-core is underlined with a dotted line, and the γ-core is with a solid line.

Peptide	Amino Acid Sequence	Net Charge at pH 7.0
	Antimicrobial Activity	Reference
OefDef1.1	**KP** **C** **TKLSKGWRGL** **C** **APHK** **C** **SSY** **C** **IHHEGAYHGA** **C** **LKNRHSKHYG** **C** **Y** **C** **YYRH** **C** **Y**	+8
	IC_50_ = 1.6 μM against *F. graminearum* (MIC = 3 μM); IC_50_ = 1.1 μM against *F. virguliforme* (MIC = 3 μM); IC_50_ = 0.4 μM against *F. oxysporum* (MIC = 1.5 μM); IC_50_ = 0.7 μM against *B. cinerea* (MIC = 1.5 μM).	[10]
OefDef1.1_V1	**KP** **C** **TKLSKGWRGL** **C** **APHK** **C** **SSY** **C** **IHHEGAYHG** **R** **C** **RG----FRRR** **C** **Y** **C** **YYRH** **C** **Y**	+10
	IC_50_ = 2−3 μM against *F. oxysporum*; IC_50_ = 2−3 μM against *B. cinerea*.	[10]
OefDef1.1_V2	**KP** **C** **TKLSKGWRGL** **C** **APHK** **C** **SSY** **C** **IHHEGAYHGA** **C** **HVRN-GKHM-** **C** **Y** **C** **YYRH** **C** **Y**	+7
	IC_50_ = 0.3−0.75 μM against *F. oxysporum*; IC_50_ = 0.7−1.5 μM against *B. cinerea*.	[10]
OefDef1.1_V3	**KP** **C** **TKLSKGWRGL** **C** **APHK** **C** **SSY** **C** **IHHEGAYHGA** **C** **AAA** **RHSKHYG** **C** **Y** **C** **YYRH** **C** **Y**	+7
	Displayed antifungal activity similar to that of the wild-type OefDef1.1.	[10]
OefDef1.1_V4	**KP** **C** **TKLSKGWRGL** **C** **APHK** **C** **SSY** **C** **IHHEGAYHGA** **C** **LKN** **AAA** **KHYG** **C** **Y** **C** **YYRH** **C** **Y**	+7
	Displayed antifungal activity similar to that of the wild-type OefDef1.1.	[10]
OefDef1.1_V5	**KP** **C** **TKLSKGWRGL** **C** **APHK** **C** **SSY** **C** **IHHEGAYHGA** **C** **LKNRHS** **AAAA** **C** **Y** **C** **YYRH** **C** **Y**	+7
	Was even more potent than the wild-type OefDef1.1.	[10]

**Table 14:** Snakins and derived peptides: sequence, net charge at pH 7.0, and antimicrobial activity. Cysteine residues are shown in red. Gaps (−) were introduced to improve the alignment. The γ-core is underlined with a solid line.

Peptide	Amino Acid Sequence	Net Charge at pH 7.0
	Antimicrobial Activity	Reference
SlSN2	**----------------------------------------** **IQTDQVSSNA---ISEGADSYK-KI** **D** ** C ** **GGA** ** C ** ** AAR ** ** C ** ** RLSSRPR-L ** ** C ** **HRA** ** C ** **GT** ** CC ** **AR** ** C ** **NCVPPGTSGNTET** ** C ** **P-** ** C ** **YASLTTHG--NKRK** ** C ** **P**	+6
	**−**	[46]
γ_48-65_SlSN2	**GA** **C** **AAR** **C** **RLSSRPR-L** **C** **HR**	+5
	IC_50_ = 4.2 μM against *C. neoformans*; IC_50_ = 23.1 μM against *C. michiganensis*; IC_50_ = 42.1 μM against *F. culmorum*; IC_50_ = 57.1 μM against *F. oxysporum*; IC_50_ = 47.5 μM against *F. solani*; IC_50_ = 152.0 μM against *F. verticilllioides*; 96% growth inhibition of *C. albicans* at 300 μM; 16% growth inhibition of *P. savastanoi* at 300 μM; 66% growth inhibition of *P. carotovorum* at 300 μM; 46% growth inhibition of *B. cinerea* at 300 μM; 31% growth inhibition of *B. sorokiniana* at 300 μM.	[46]
SlSN9	**QDSIIDLKEVEEDKQQHVGLSQALRVFTRGANRRLVQDIVLKVAKYLNNGDIALAPAPAPPPSPL** **D** ** C ** **GGL** ** C ** ** KYR ** ** C ** ** SLHSRPN-V ** ** C ** **FRA** ** C ** **GT** ** CC ** **VR** ** C ** **KCVPPGTFGNREK** ** C ** **GK** ** C ** **YTEMTTHG--NKTK** ** C ** **P**	+8
	**−**	[46]
γ_89-106_SlSN9	**GL** **C** **KYR** **C** **SLHSRPN-V** **C** **FR**	+4
	IC_50_ = 5.1 μM against *C. neoformans*; IC_50_ = 24.0 μM against *C. michiganensis*; IC_50_ = 42.4 μM against *F. culmorum*; IC_50_ = 138.8 μM against *F. solani*; IC_50_ = 99.8 μM against *F. verticilllioides*; 96% growth inhibition of *C. albicans* at 300 μM; 100% growth inhibition of *P. savastanoi* at 300 μM; 58% growth inhibition of *P. carotovorum* at 300 μM; 58% growth inhibition of *F. oxysporum* at 300 μM; 60% growth inhibition of *B. cinerea* at 300 μM; 33% growth inhibition of *B. sorokiniana* at 300 μM.	[46]
SlSN10	**-----------------------------------------** **LQEVISGKP---PAPSPQPPK-PI** **D** ** C ** **TGS** ** C ** ** KTR ** ** C ** ** SKSSRQN-L ** ** C ** **NRA** ** C ** **GS** ** CC ** **RT** ** C ** **HCVPPGTSGNYEA** ** C ** **P-** ** C ** **YFNLTTHN--STRK** ** C ** **P**	+7
	**−**	[46]
γ_47-64_SlSN10	**GS** **C** **KTR** **C** **SKSSRQN-L** **C** **NR**	+5
	IC_50_ = 126.7 μM against *F. culmorum*; IC_50_ = 43.8 μM against *F. oxysporum*; 96% growth inhibition of *C. neoformans* at 300 μM; 90% growth inhibition of *C. albicans* at 300 μM; 94% growth inhibition of *C. michiganensis* at 300 μM; 18% growth inhibition of *P. savastanoi* at 300 μM; 65% growth inhibition of *P. carotovorum* at 300 μM; 39% growth inhibition of *B. cinerea* at 300 μM; 22% growth inhibition of *B. sorokiniana* at 300 μM; no activity against *F. verticillioides* at 300 μM.	[46]
TkSN1	**--------------------------------------------------------------** **ASG** **F** ** C ** **AGK** ** C ** ** AVR ** ** C ** ** ARSRAKRGA ** ** C ** **MKY** ** C ** **GL** ** CC ** **EE** ** C ** **ACVPTGRSGSRDE** ** C ** **P-** ** C ** **YRDMLTAGPRKRPK** ** C ** **P**	+9
	**−**	[53]
TkSN1_39-57_	**GK** **C** **AVR** **C** **ARSRAKRGA** **C** **MK**	+7
	IC_50_ = 6.0 μM against *C. neoformans* (100% germination inhibition at 20 μM); 98% germination inhibition of *C. albicans* at 300 μM; IC_50_ = 12.0 μM against *C. michiganensis* (100% germination inhibition at 50 μM); IC_50_ = 27.5 μM against *F. culmorum* (80% germination inhibition at 150 μM); IC_50_ = 93.6 μM against *F. oxysporum* (68% germination inhibition at 150 μM); 54% germination inhibition of *F. solani* at 300 μM; 18% germination inhibition of *P. savastanoi* at 180 μM; 59% germination inhibition of *P. carotovorum* at 300 μM; no activity against *F. verticillioides* at 300 μM.	[53]

**Table 15:** nsLTPs and derived peptides: sequence, net charge at pH 7.0, and antimicrobial activity. Cysteine residues are shown in red. Gaps (−) were introduced to improve the alignment. The γ-core is underlined with a solid line.

Peptide	Amino Acid Sequence	Net Charge at pH 7.0
	Antimicrobial Activity	Reference
SlLTPg2.4	**-----------** **AQLS** **SD** ** C ** **TNVL-----VSMSP** ** C ** **LNYITGN-SSSSPSSG** ** CC ** ** TQLGTVVKNNPE ** ** C ** **L** ** C ** **QVLNGGGS--NMGL** **NINQTQALALPNA** ** C ** **KVQT-PSISK** ** C ** **NAGSPTSSPAGTPSSPNTGGSGSGSIPSSRDASNDASLTKMIDLPFFLILFISSYASAFMA**	0
	**−**	[46]
γ_56-72_SlLTPg2.4	**SG** **CC** **TQLGTVVKNNPE** **C**	0
	8% growth inhibition of *C. albicans* at 300 μM; 4% growth inhibition of *C. michiganensis* at 300 μM; 2% growth inhibition of *P. savastanoi* at 300 μM; 4% growth inhibition of *P. carotovorum* at 300 μM; no activity against *C. neoformans, F. culmorum, F. oxysporum, F. solani, F. verticillioides, B. cinerea* and *B. sorokiniana* at 300 μM.	[46]
SlLTPg2.5	**------------QESDD** ** C ** **TNVW-----VSMSP** ** C ** **LNYYVD--STSPQFSG** ** CC ** ** TQLSTVVDEKSE ** ** C ** **L** ** C ** **QVLK------DLG** **LNINQTRLSALTTA** ** C ** **KVQT-PPASN** ** C ** **NGRGSASQGGPNDATSTNMAAPFSFFFLLIASYASIINIT**	−4
	**−**	[46]
γ_53-69_SlLTPg2.5	**SG** **CC** **TQLSTVVDEKSE** **C**	−2
	19% growth inhibition of *C. neoformans* at 300 μM; 13% growth inhibition of *C. albicans* at 300 μM; 8% growth inhibition of *C. michiganensis* at 300 μM; 20% growth inhibition of *P. savastanoi* at 300 μM; 5% growth inhibition of *P. carotovorum* at 300 μM; no activity against *F. culmorum, F. oxysporum, F. solani, F. verticillioides, B. cinerea* and *B. sorokiniana* at 300 μM.	[46]
SlLTPg2.8	**QDSPPAPEAPAPSPGVD** ** C ** **FRVL-----VNMSD** ** C ** **LAFVERGSNTTTPGKG** ** CC ** ** PEIAGLLDSNPI ** ** C ** **L** ** C ** **HMLGRAHSGAKIG** **FNIDVDKALKLPSA** ** C ** **SLEF-PPSTT** ** C ** **SDLGIPVGAPLPSEESPAPSPGKQTLAVFSVLF**	−5
	**−**	[46]
γ_70-86_SlLTPg2.8	**KG** **CC** **PEIAGLLDSNPI** **C**	−1
	54% growth inhibition of *C. neoformans* at 300 μM; 2% growth inhibition of *C. albicans* at 300 μM; 15% growth inhibition of *P. savastanoi* at 300 μM; 5% growth inhibition of *P. carotovorum* at 300 μM; 22% growth inhibition of *B. sorokiniana* at 300 μM; no activity against *C. michiganensis, F. culmorum, F. oxysporum, F. solani, F. verticillioides, B. cinerea* at 300 μM.	[46]
TkLTP2.25	**--------------AAG** ** C ** **DASA-------LSP** ** C ** **VGAIMVG---GAVTPG** ** CC ** ** ARLRAQRA---- ** ** C ** **L** ** C ** **QYAREP----SYR** **GYVNSPRAQSVVAA** ** C ** **GLPR----PK** ** C **	+6
	**−**	[53]
TkLTP2.25_50-62_	**PG** **CC** **ARLRAQRA----** **C**	+3
	IC_50_ = 45.0 μM against *C. neoformans* (100% germination inhibition at 100 μM); 13% germination inhibition of *C. albicans* at 300 μM; IC_50_ = 94.6 μM against *C. michiganensis* (95% germination inhibition at 300 μM); 36% germination inhibition of *F. culmorum* at 300 μM; 34% germination inhibition of *F. oxysporum* at 300 μM; 25% germination inhibition of *P. savastanoi* at 300 μM; 8% germination inhibition of *P. carotovorum* at 300 μM; no activity at 300 μM against *F. solani* and *F. verticillioides*.	[53]
TkLTPd5.6	**--------------AGE** ** C ** **GKTPADKMALKLAP** ** C ** **ASAGQDP--KSAPSSG** ** CC ** ** TAVHTIGKQSPK ** ** C ** **L** ** C ** **AVMLSDT---AKS** **AGIKPEVAMSIPKR** ** C ** **NLVDRPVGYK** ** C ** **GAYTLP**	+6
	**−**	[53]
TkLTPd5.6_59-75_	**SG** **CC** **TAVHTIGKQSPK** **C**	+2
	11% germination inhibition of *C. neoformans* at 300 μM; 24% germination inhibition of *C. albicans* at 300 μM; 23% germination inhibition of *C. michiganensis* at 300 μM; 18% germination inhibition of *P. savastanoi* at 300 μM; 2% germination inhibition of *P. carotovorum* at 300 μM; no activity at 300 μM against *F. culmorum*, *F. oxysporum*, *F. solani*, *F. verticillioides*.	[53]

**Table 16:** SlMEG2, thionin TkThi1, knottin Tk-AMP-K2, and derived peptides: sequence, net charge at pH 7.0, and antimicrobial activity. Cysteine residues are shown in red. The γ-core is underlined with a solid line.

Peptide	Amino Acid Sequence	Net Charge at pH 7.0
	Antimicrobial Activity	Reference
SlMEG2	**APISQAKGSEMVPLIEPGKAEKMMIMLNNTRRKLGSFQI** ** C ** **AL** ** C ** **T** ** CC ** **GG** **KAV** ** C ** **LPTP** ** CC ** **YAIN** ** C ** **NIPNRPFGY** ** C ** ** SFTPKT ** ** C ** **N** ** C ** **FGCHY**	+6
	**−**	[46]
γ_92-104_SlMEG2	**RPFGY** **C** **SFTPKT** **C**	+2
	97% growth inhibition of *C. neoformans* at 300 μM; 92% growth inhibition of *C. michiganensis* at 300 μM; 16% growth inhibition of *P. savastanoi* at 300 μM; 40% growth inhibition of *B. cinerea* at 300 μM; 36% growth inhibition of *B. sorokiniana* at 300 μM; no activity against *C. albicans, P. carotovorum, F. culmorum, F. oxysporum, F. solani, F. verticillioides* at 300 μM.	[46]
TkThi1	**GW** ** C ** **DRA** ** C ** **LFQ** ** C ** **THSGGQEDR** ** C ** **RTF** ** C ** **R** ** C ** **PNKSGERNALEL** ** C ** **TSG** ** C ** **SSSI** ** C ** **GIINTVDGTEAGKHAAVGR** ** C ** ** NEA ** ** C ** ** ASF ** ** C ** **SKGEHGIQSVAT**	0
	**−**	[53]
TkThi1_96-109_	**VGR** **C** **NEA** **C** **ASF** **C** **SK**	+1
	13% germination inhibition of *C. albicans* at 300 μM; 40% germination inhibition of *C. michiganensis* at 300 μM; 7% germination inhibition of *P. carotovorum* at 300 μM; no activity at 300 μM against *C. neoformans*, *F. culmorum*, *F. oxysporum*, *F. solani, F. verticillioides* and *P. savastanoi*.	[53]
Tk-AMP-K2	**PG** ** C ** **PVGQLMK** ** C ** ** RTTFP ** ** CC ** ** G ** **GR** ** C ** **VY** ** C **	+3
	**−**	[53]
Tk-AMP-K2_10-23_	**K** **C** **RTTFP** **CC** **GGR** **C** **V**	+3
	100% germination inhibition of *C. neoformans* at 300 μM; 49% germination inhibition of *C. albicans* at 300 μM; 71% germination inhibition of *C. michiganensis* at 150 μM; 42% germination inhibition of *F. culmorum* at 300 μM; 11% germination inhibition of *F. oxysporum* at 300 μM; 34% germination inhibition of *F. solani* at 150 μM; 34% germination inhibition of *F. verticillioides* at 300 μM; 85% germination inhibition of *P. savastanoi* at 300 μM; 70% germination inhibition of *P. carotovorum* at 300 μM.	[53]

**Table 17:** WAMP-2 and derived peptides: sequence, net charge at pH 7.0, and antimicrobial activity. Cysteine residues are shown in red. The γ-core is underlined with a solid line.

Peptide	Amino Acid Sequence	Net Charge at pH 7.0
	Antimicrobial Activity	Reference
WAMP-2	**AQR** ** C ** **GDQARGAK** ** C ** **PN** ** C ** **L** ** C ** ** C ** ** GKYGF ** ** C ** ** GSGDAY ** ** C ** **GKGS** ** C ** **QSQ** ** C ** **RG** ** C ** **R**	+5
	IC_50_ = 6.6 μM against *B. sorokiniana*; IC_50_ = 23.0 μM against *A. alternata*; IC_50_ = 8.0 μM against *Cladosporium cucumerinum*; IC_50_ = 8.8 μM against *F. oxysporu*m; IC_50_ = 52.9 μM against *F. culmorum*.	[54]
WAMP-N	**AQR** ** C ** **GDQARGAK** ** C **	+2
	IC_50_ = 53.5 μM against *B. sorokiniana* (80.8% inhibition at 200 μg/mL); IC_50_ = 75.3 μM against *A. alternata* (77.2% inhibition at 200 μg/mL); IC_50_ = 205.5 μM against *C. cucumerinum* (43.6% inhibition at 200 μg/mL); IC_50_ = 174.6 μM against *F. oxysporum* (44.9% inhibition at 200 μg/mL); IC_50_ = 243.8 μM against *F. culmorum* (45.0% inhibition at 200 μg/mL); IC_50_ > 500 μM against *F. avenaceum* (40.4% inhibition at 400 μg/mL); IC_50_ = 161.5 μM against *Parastagonospora nodorum* (63.5% inhibition at 400 μg/mL).	[54]
WAMP-G1	**L** **CC** **GKYGF** **C** **GSG**	+1
	IC_50_ = 228.7 μM against *B. sorokiniana* (45.7% inhibition at 200 μg/mL); IC_50_ > 500 μM against *A. alternata* (16.7% inhibition at 200 μg/mL); IC_50_ > 500 μM against *C. cucumerinum* (19.2% inhibition at 400 μg/mL); IC_50_ > 500 μM against *F. oxysporum* (16.7% inhibition at 400 μg/mL); IC_50_ > 500 μM against *F. culmorum* (6.5% inhibition at 400 μg/mL); IC_50_ > 500 μM against *P. nodorum* (28.5% inhibition at 400 μg/mL); no activity against *F. avenaceum* at 400 μg/mL	[54]
WAMP-G2	**CC** **GKYGF** **C** **GSGDAY** **C**	0
	IC_50_ = 127.3 μM against *B. sorokiniana* (54.3% inhibition at 200 μg/mL); IC_50_ = 94.9 μM against *A. alternata* (61.1% inhibition at 200 μg/mL); IC_50_ = 267.4 μM against *C. cucumerinum* (23.6% inhibition at 200 μg/mL); IC_50_ = 255.1 μM against *F. oxysporum* (35.5% inhibition at 200 μg/mL); IC_50_ > 500 μM against *F. culmorum* (26.2% inhibition at 400 μg/mL); IC_50_ = 393.1 μM against *F. avenaceum* (17.5% inhibition at 200 μg/mL); IC_50_ = 276.5 μM against *P. nodorum* (24.1% inhibition at 200 μg/mL).	[54]
WAMP-C	**GKGS** **C** **QSQ** **C** **RG** **C** **R**	+3
	IC_50_ = 313.6 μM against *B. sorokiniana* (48.7% inhibition at 400 μg/mL); IC_50_ = 401.9 μM against *A. alternata* (45.1% inhibition at 400 μg/mL); IC_50_ = 3.9 μM against *C. cucumerinum* (70.2% inhibition at 50 μg/mL); IC_50_ > 500 μM against *F. oxysporum* (11.5% inhibition at 400 μg/mL); IC_50_ > 500 μM against *F. culmorum* (23% inhibition at 400 μg/mL); IC_50_ = 240.7 μM against *P. nodorum* (59.9% inhibition at 400 μg/mL); no activity against *F. avenaceum* at 400 μg/mL.	[54]

## Data Availability

Not applicable.

## Supplementary Materials

The following supporting information can be downloaded at: https://www.mdpi.com/article/10.3390/ijms24010483/s1.
