# Supplementary figures and images for: The γ-Core Motif Peptides of Plant AMPs as Novel Antimicrobials for Medicine and Agriculture
Source: Int J Mol Sci. 2022 Dec 28;24(1):483. doi: 10.3390/ijms24010483 (PMC9820530; doi:10.3390/ijms24010483)

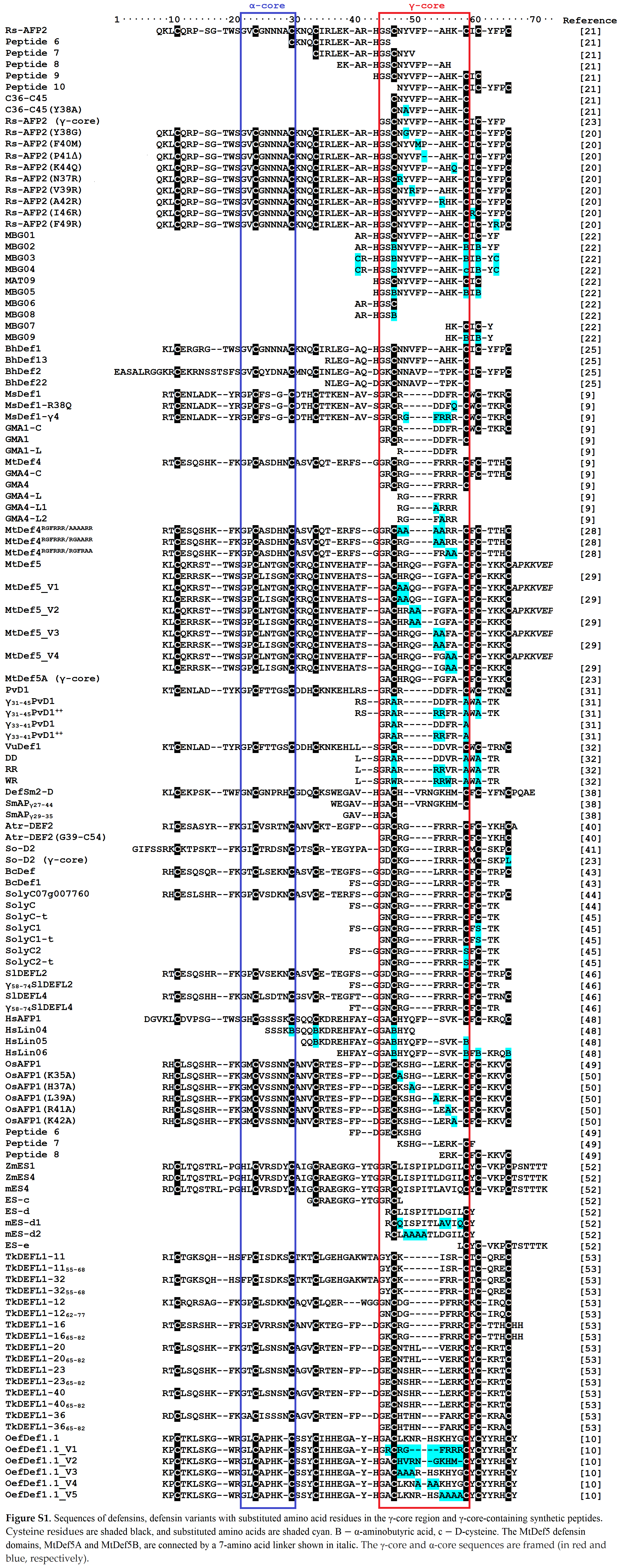

Supplement: Supplementary file 1 [file ijms-24-00483-s001.zip › Figure S1.tif]
